# Agricultural land use shapes short and long‑term bacterial diversity, community structure, and assembly in biofilms of adjacent streams

**DOI:** 10.1186/s40793-025-00837-9

**Published:** 2025-12-20

**Authors:** Rubén Martínez-Cuesta, Rebecca Hoess, Sebastian Floßmann, Juergen Geist, Michael Dannenmann, Michael Schloter, Stefanie Schulz

**Affiliations:** 1 Chair of Environmental Microbiology, TUM School of Life Sciences, Technical University of Munich, Emil-Ramann-Straße 2, 85354 Freising, Germany; 2 Research Unit Comparative Microbiome Analysis, Helmholtz Zentrum Munich, Ingolstädter Landstraße 1, 85764 Neuherberg, Germany; 3 Aquatic Systems Biology Unit, Technical University of Munich, Mühlenweg 22, 85354 Freising, Germany; 4 Institute of Meteorology and Climate Research - Atmospheric Environmental Research (IMK-IFU), Karlsruhe Institute of Technology, Kreuzeckbahnstraße 19, 82467 Garmisch-Partenkirchen, Germany

**Keywords:** Freshwater biofilms, Land use, 16S rRNA gene amplicon sequencing, Bacterial community assembly, Co-occurrence networks

## Abstract

**Background:**

Intensive agriculture can disrupt adjacent stream ecosystems by increasing nutrient runoff, suspended and dissolved organic matter, and pesticide loads. Freshwater biofilms are surface-attached microbial communities that host complex interaction networks and play a key role in nutrient cycling and bioremediation. If adjacent land use drastically shifts microbial community composition and assembly, the functional resilience and adaptive capacity of biofilms under changing conditions may be impaired. In this study, we compared developing and mature biofilm samples from three sites along the Otterbach and two sites along the Perlenbach stream (Bavaria, Germany). The Otterbach sites, located in an area with agriculture and a nearby village, were adjacent to an intensively managed cropland, an extensively managed grassland, and a forest, while the Perlenbach flows through an area free of cropping and fertilization, with sites adjacent to an extensively managed grassland and a forest. Bacterial community composition was assessed through 16S rRNA gene amplicon sequencing. Bacterial diversity, differential abundance, community assembly and co-occurrence network analyses were performed.

**Results:**

Adjacent intensive land use increased bacterial alpha diversity in both developing and mature biofilms. Moreover, adjacent intensive and extensive land use shaped bacterial community composition and increased the relevance of deterministic processes in bacterial community assembly, especially in developing biofilms, increasing the presence of key responding taxa such as *Arenimonas*, *Blastocatella*, *Gemmatimonas*, *Flectobacillus*, *Leptothrix*, *Flavobacterium* or *Rhodoferax*. These taxa were also detected in the co-occurrence networks of agriculturally influenced sites, displaying strong connectivity and centrality. These effects were limited to the Otterbach stream, which exhibited higher overall nutrient concentrations.

**Conclusions:**

Agricultural land use strongly influenced bacterial richness, composition, and assembly in biofilms from adjacent stream ecosystems, particularly in developing biofilms from the most anthropogenically impacted stream, driven by the proliferation of key responding taxa. This showcased how anthropogenic nutrient inputs can redirect biofilm development pathways and potentially alter the ecological role of biofilms in stream ecosystems.

**Supplementary Information:**

The online version contains supplementary material available at 10.1186/s40793-025-00837-9.

## Background

Land use and land use intensity are amongst the strongest drivers that can affect species composition, diversity and ecosystem services [1], not only altering adjacent terrestrial ecosystems [2], but also diversity and ecosystem functions in adjacent aquatic environments [3, 4]. In particular, agricultural land use can increase nutrient deposition rates, dissolved and suspended organic matter, and pollutants [5], which can deteriorate water quality and subsequently, impair the properties, biodiversity, structure and functioning of aquatic ecosystems [6].

Such effects have been well studied in freshwater fine sediment microbial communities [7, 8]. These studies have identified a decline in microbial richness with increasing land use intensity and an increased abundance of microbial taxa associated with nutrient turnover (e.g., *Gemmatimonadaceae*) [7], pesticide degradation (e.g., *Phenylobacterium)* [7], or the breakdown of algal cell components following algal proliferation (e.g., *Latescibacteria*) [9]. The effect sizes have been shown to increase over time, implying cumulative impacts of land use on sediment microbial communities [10]. Thus, sediments are often described as an “archive” of environmental change in freshwater ecosystems [11].

In contrast, the influence of different adjacent land use types, in particular agricultural land use, on more dynamic and rapidly responding components of freshwater ecosystems, such as epilithic biofilms growing on the surface of hard and stable substrates like rocks or bedrocks, remains poorly understood, despite their potential to affect ecosystem resilience and functionality. These effects could expand through the food web, for example by reducing resources for grazers or altering nutrient cycling[12, 13].

Freshwater biofilms, defined as the complex mixture of phototrophic and heterotrophic communities which are attached to submerged substrates [14–16], play a key role in primary production, nutrient cycling, and as a food source for many freshwater organisms [16, 17]. In addition, they provide relevant ecosystem services such as phosphorus absorption [18] or bioremediation of xenobiotics (e.g. pesticides) from the water [19], and shifts in their microbial community composition can be used as an indicator of pollution [20]. Therefore, freshwater biofilms have been considered as ideal candidates for monitoring the ecological effects of different land use types on the condition of aquatic environments [21–24], which included the assessment of the effects of agricultural pesticides, the impact of grassland degradation on nearby streams [25], or the identification of direct indicators to predict land use intensity [23].

Microbial community assembly in freshwater biofilms involve the effective surface colonization of cells from the surrounding environment, where they constitute the community within a matrix [26, 27]. However, under environmental stress, community diversity and functional redundancy may decline, potentially leading to a reduced capacity of biofilms to perform key ecosystem functions. In freshwater ecosystems, microbial community assembly is driven by a combination of deterministic processes [28] (such as environmental selection), which reflect how abiotic and biotic factors shape community structure, and stochastic processes, which involve random events such as drift (random birth–death events) or dispersal limitation [29, 30]. Given the role of priority effects in the colonization of developing biofilms [31, 32], where early-arriving taxa may not necessarily be stress-tolerant or capable of forming mutualistic, and syntrophic interactions [33], the assembly of these initial communities can be highly susceptible to environmental filters. Therefore, we hypothesized that adjacent agricultural land use will exert a significant impact on developing biofilms in terms of richness and community composition, increasing the relevance of deterministic processes in bacterial community assembly, especially in developing biofilms. To this end, we evaluated bacterial community composition, assembly, and interaction through metabarcoding with 16S rRNA gene amplicon sequencing of developing versus mature biofilms along two streams that flow through different land-use types and differ in nutrient input.

## Methods

### Sites description and sampling

**Fig. 1:**
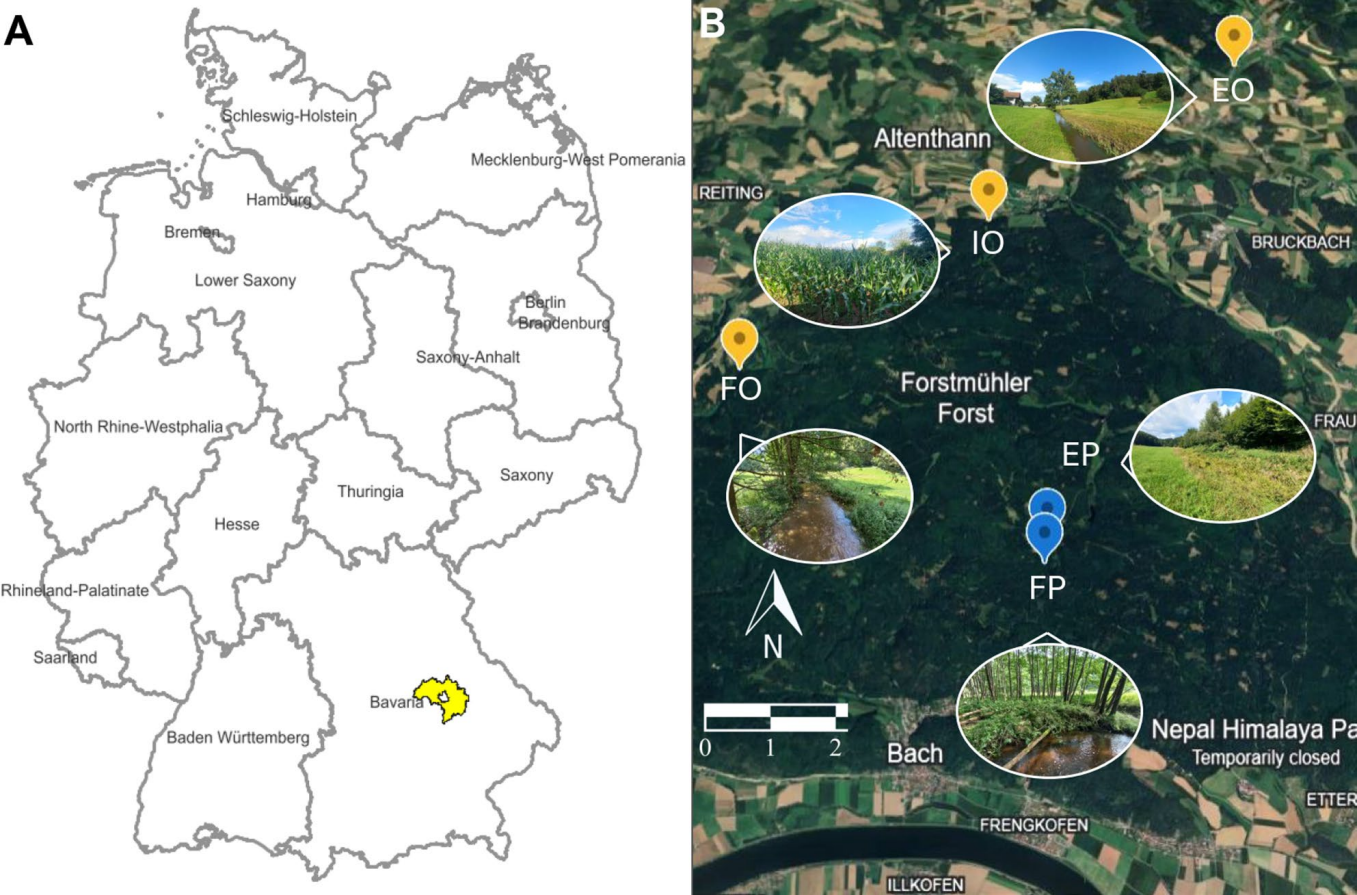
Overview of: **A** study area in Germany where the experiment was carried out (in yellow), sourced from https://geojson.io; **B** satellite image from Google Earth (https://earth.google.com), of the three sites from the Otterbach (in yellow) and the Perlenbach stream (in blue), which ultimately merge with the Danube River. In the Otterbach, EO stands for the extensive grassland site, IO for the intensive farming site and FO for the forest site. In the Perlenbach, EP stands for the extensive grassland site and FP for the forest site

Biofilm samples were collected at two time points in 2023 (August and November) along two streams in the Falkensteiner Vorwald area of the Bavarian Forest (Bavaria, Germany). The Otterbach stream undergoes higher anthropogenic pressure due to agriculture, the presence of a village and a wastewater treatment plant, while the Perlenbach represents the reference conditions of low mountain streams of this region, with neither fertilization nor cropping taking place in the surrounding area. The Otterbach stream has an average annual discharge of 0.833 m^3^/s. Three sites were sampled along a land use gradient ranging from an extensively managed grassland (EO, fertilized twice per year, mowed once per year) located below an upstream village (49º06′09″ N 12º21′30″ E—473 m), a conventional intensive farming site with corn (*Zea mays*) plantations (IO, 49°04′51″ N 12°18′52″ E – 443 m, respectively), and a coniferous forest site composed mainly of *Picea abies* and *Sambucus nigra* (FO, 49°03′45″ N 12°16′29″ E – 378 m) within the riparian zone (Fig. 1). Two sites were sampled along the Perlenbach stream (Fig. 1), one adjacent to an extensively managed grassland (EP, 49°02′34″ N 12°19′22″ E – 486 m, not fertilized, mowed once per year) followed by a site with closed riparian vegetation covered by elder trees (*Sambucus nigra*) lying within a pine forest (FP, 49°02′24″N 12°19′20″E – 502 m). Both streambeds are derived from the same parent material, likely granite, and are rich in silicate minerals [34].

Based on previous work assessing the effects of different sampling methodologies and exposure times for stream biofilms[35], coupon sampling devices with glass slides (150 mm × 20 mm; Fig. S1) were installed and exposed for four weeks at the sampling sites. These slides captured developing biofilms (DB), allowing the assessment of short-term adjacent land use effects on biofilm bacterial communities. Additionally, mature biofilms (MB) were sampled from 150 mm × 20 mm of the surface area of natural hard substrates such as large rocks collected at the sampling sites, to account for long-term adjacent land use effects on biofilm bacterial communities.

Sterile toothbrushes were used to collect five biofilm replicates from both DB and MB from the substratum surface from each site. DB and MB samples were transported in river water collected from the sites and wrapped in tin foil to prevent degradation from sunlight and temperature changes. The tubes containing DB and MB samples were centrifuged at 500 rpm for 10 min, the supernatants were discarded, and the remaining pellets were frozen at −20 °C until DNA extraction.

### Abiotic parameters and physicochemical properties

Physicochemical properties were measured in situ at each sampling location from a depth of 5 cm below the water surface. These included dissolved oxygen (mg/L), temperature (°C), electrical conductivity (µS/cm), and pH, which were measured using a handheld Multi 3430 G meter (WTW, Weilheim, Germany). Redox potential (mV) was measured following established protocols [36] using a handheld pH 3110 m (WTW, Weilheim, Germany). Turbidity (NTU) was measured from three 10 mL replicates using a TURB 355 T handheld turbidimeter (WTW, Weilheim, Germany). Stream depth (cm) and width were measured using a measuring rod and tape, and flow velocity (m/s) was measured at both the stream bottom and surface using a Flowtherm NT flow meter (Höntzsch, Waiblingen, Germany). Water samples for chemical analysis were filtered on site through 0.45 µm nylon filters (Minisart HighFlow PES, Sartorius AG, Germany). Concentrations (mg/L) of chloride, nitrate, nitrite, phosphate, sulfate, ammonium, magnesium, and calcium were determined using two Dionex ICS-1100 ion chromatographs (Thermo Fisher Scientific, Dreieich, Germany). Anions were separated using AG-23/AS-23 guard/separation columns with a disodium carbonate/sodium hydrogen carbonate eluent, while cations were separated on CG-16/CS-16 columns with a methane sulfonic acid eluent. Furthermore, continuous measurements of DOC concentrations were conducted at the forest and intensive farming site of Otterbach from August to November using a UV/Vis spectrometer probe (spectro::lyser, s::can Messtechnik GmbH, Vienna, Austria) according to Jacobs et al., [37].

### DNA extraction, library preparation and sequencing

Total DNA was extracted from biofilm pellets using the NucleoSpin® Soil Kit (Macherey–Nagel, Düren, Germany) following the manufacturer’s guidelines. The SL1 buffer was used for the chemical cell lysis, and the Precellys24 homogenizer (Bertin Technologies, Montigny-le-Bretonneux, France) was used for the mechanical cell lysis. Bead beating tubes with kit reagents and without sample were used as negative extraction controls. 30 μL of diethyl pyrocarbonate (DEPC) water was used to elute the extracted DNA. DNA sample concentrations were measured using the Qubit dsDNA HS assay kit (Thermo Fisher Scientific, Bremen, Germany). DNA size and quality were checked with a NanoDrop ® ND-1000 system (Thermo Fisher Scientific, Bremen, Germany) and electrophoreses in 1.5% (w/v) agarose gels.

For the exploration of the biofilm bacterial communities and their diversity, we followed the quality guidelines of the 16S rRNA Metagenomic Sequencing Library Preparation protocol (Illumina, San Diego, CA, USA). Briefly, 5 ng of DNA extracts were amplified using the primer pair for bacteria 008F (5′-AGAGTTTGATCMTGGC-3′) and 343R (5’-CTGCTGCCTYCCGTA-3’), which target the V1-V2 region of the 16S rRNA gene [38]. Preliminary tests indicated a reduced off-target amplification when compared to the typically used 515F/806R primer set (data not shown).

Each PCR reaction consisted of 25 µL which contained 12.5 µL of NEB Next High-Fidelity Master Mix (Thermo Fisher Scientific, Bremen, Germany), 0.5 µL of each primer at 10 pmol/µl, 2.5 µL of 3% BSA, 1 µl of the template DNA and 8 µL of DEPC water. The PCR thermal profile was as follows: 98 °C for 1 min, followed by 25 cycles of 98 °C for 10 s, 55 °C for 30 s and 72 °C for 30 s, and a final extension step of 72 °C for 5 min. Samples were indexed using the Nextera® XT Index Kit v2 (Illumina, USA) and purified with MagSi-NGSprep Plus Beads (ratio 0.6 beads: 1 sample). The quality of the samples was assessed with a 5200 Fragment Analyzer System (Agilent Technologies, Santa Clara, USA). Each library was diluted to 4 nM and sequenced with the MiSeq Reagent kit v3 (600 cycles) and the Illumina MiSeq (Illumina, San Diego, CA, USA) device for paired-end sequencing (PE300; 2 × 300 bp).

### Bioinformatic processing

Sequencing adapters were trimmed off from the raw sequences using fastp Chen et al., [39] v0.23.4 with default settings. Within R v4.3.1, the remaining sequences were subsequently processed with DADA2 [40] v1.28.0 until the obtention of amplicon sequencing variants (ASVs) (Table S1). The following parameters were selected throughout the process: forward and reverse reads were trimmed to 263 and 201 bp, respectively, where the quality score dropped below 30. Additionally, 16 and 15 nucleotides were trimmed from the start of the forward and reverse reads, respectively, to remove the primers. The resulting ASVs were taxonomically classified using a trained classifier from the SILVA [41] v138.2 database at the 99% sequence identity using the IDTAXA [42] algorithm within the DECIPHER R package [43] v2.28.0. A phylogenetic tree was created aligning the sequences of the ASVs table with MAFFT [44] and then using IQ-TREE2 [45].

The output of the taxonomic assignation and the phylogenetic tree were exported into the phyloseq v1.38.0 R package. 74 ASVs from the negative controls were classified as contaminants when establishing a prevalence threshold of 0.05 and were subsequently removed using the decontam [46] v1.12 package. ASVs classified as mitochondrial and chloroplast sequences were removed from the dataset using the *subset_taxa* function of phyloseq. To assess whether a complete coverage of the bacterial communities was achieved with the selected sequencing depth, rarefaction curves were plotted using the ASVs table and the *rarecurve* function of the vegan v2.6–4 R package (Fig. S2). Sampling depth differences were corrected with the cumulative sum scaling (CSS) method using the *normalize* function of the microbiomeMarker v1.9.0 R package [47].

### Statistical and ecological analyses

The observed ASVs per sample were determined using the *alpha* function of the microbiome v1.22.0 package. To assess bacterial richness differences between adjacent land use type within sample types, Wilcoxon rank-sum tests were carried out using the *stat_compare_means* function of the ggpubr v0.6.0 R package. *P* values were adjusted for multiple comparisons with the false discovery rate (FDR) method using the *p.adjust.methods* function.

Principal Coordinate Analyses (PCoA) were carried out using the Bray–Curtis statistic to quantify samples dissimilarity, the ordinate function of the phyloseq package and visualized with the *plot_ordination* function. Nutrient and abiotic parameter datasets were fitted onto the PCoA ordinations using the *env_fit* function from the vegan package. Only variables with a permutation test *p*-value < 0.05 were included in the plots. Additionally, PERMANOVA tests were conducted using the *adonis2* function of the vegan package to assess the effects of land use type, sampling time and their interaction on dissimilarity within each sample type. The β-diversity variation within samples from the same site, sampling time and sample type was assessed using the *betadisper* function of the vegan package. Differences in distance to centroid were assessed with the Kruskal–Wallis test first, followed by post hoc with pairwise Wilcoxon rank-sum tests with *P* values corrected for multiple testing using the FDR method.

To detect enriched taxa associated to each land use type, we deployed the Analysis of Composition of Microbiomes with bias correction (ANCOM-BC2) method through the ancombc2 [48] R package v2.2.2 comparing the ASVs from the forest sites to the ones from the extensive grassland and the intensive agricultural site. The *p*-value threshold was set to 0.05 and we selected log fold change minimum thresholds of 0.5 and -0.5. Results were plotted using ggplot2 v3.5.1.

To assess the effect of the adjacent land use types on the relative importance of ecological processes driving bacterial community assembly, we deployed the phylogenetic bin-based null model using the iCAMP v1.5.12 R package. Briefly, ASVs were fitted into different bins based on their phylogenetic distance, and the contribution of each bin to the different ecological processes was calculated. The general threshold for phylogenetic distance (ds) was set to 0.2 and the bin size limit to 12 following the recommendations [49].

To further investigate the ecological functions of the overlapping bacterial taxa identified in the differential abundance and community assembly analyses, we constructed bacterial co-occurrence networks using the NetCoMi [50] v1.1.0 R package and the SPIEC-EASI [51] (SParse InversE Covariance Estimation for Ecological Association Inference) method. For this analysis, we kept ASVs present in at least 20% of the samples and with a relative abundance higher than 0.01. The edge selection was performed using t-tests as sparsification method and the local false discovery rate as the multiple testing correction approach. Clusters of co-occurring ASVs were identified using the *cluster_fast_greedy* function.

## Results

### Physicochemical properties

In agreement with the differences in land use, the Otterbach and Perlenbach streams also differed in terms of their physicochemical properties at the two sampling times (Table 1). Electric conductivity (EC) differed substantially between the two streams, reflecting differences in ion concentrations. EC values ranged from 234 to 275 µS/cm in the Otterbach and from 89 to 96 µS/cm in the Perlenbach. Of those ions, Ca^2+^, Cl^−^, Na^+^ and particularly NO_3_^−^ showed the biggest variation between streams (Table 1). Phosphate (PO_4_^3−^) was only detected at the forest site of the Otterbach at the autumn sampling. Sulphate (SO₄^2^-) concentrations remained relatively stable within streams, but were generally higher in the Otterbach compared to the Perlenbach.

**Table 1:** Average (N = 3) of physicochemical properties of the open water measured at each sampling site along the Otterbach and Perlenbach streams at the first (T1) and second (T2) sampling time: extensive grassland (EO), intensive farming (IO) and forest (FO), and from the Perlenbach sites: extensive grassland (EP) and forest (FP)

Physicochemical Properties	Otterbach						Perlenbach			
	EO		IO		FO		EP			FP
	T1	T2	T1	T2	T1	T2	T1	T2	T1	T2
O_2_ (mg/L)	10.21	10.48	9.10	11.25	9.58	10.58	8.77	10.50	8.97	9.08
T (°C)	14.0	8.0	15.0	8.2	15.8	9.2	15.2	8.8	14.4	9.7
pH	7.30	7.02	7.30	7.60	7.70	7.35	7.00	7.10	7.00	7.64
EC (µS/cm)	275	241	288	234	264	264	91	89	92	96
Turb (NTU)	8.91	16.67	9.87	26.94	11.91	9.41	8.22	22.53	5.25	22.08
Ca^2+^ (mg/L)	24.59	22.40	25.16	24.34	23.15	22.02	9.28	8.11	9.18	8.58
Cl^−^ (mg/L)	32.30	25.76	33.87	29.21	29.19	25.79	5.63	2.70	4.09	2.50
K^+^ (mg/L)	2.54	2.55	1.96	3.05	2.33	3.43	0.69	1.48	0.27	1.45
Mg^2+^ (mg/L)	3.66	3.60	4.14	3.89	3.96	3.56	1.72	1.51	1.73	1.73
Na^+^ (mg/L)	23.01	16.61	20.89	18.00	20.62	16.55	9.03	4.76	8.48	5.38
NH_4_^+^ (mg/L)	0.00	0.22	0.00	0.23	0.02	0.00	0.23	0.08	0.00	0.00
NO_2_^−^ (mg/L)	0.00	0.00	0.00	0.05	0.00	0.04	0.01	0.02	0.00	0.00
NO_3_- (mg/L)	16.81	21.5	20.57	20.13	17.66	17	1.91	1.60	0.93	1.81
PO₄^3^⁻ (mg/L)	0.00	0.00	0.00	0.00	0.00	0.28	0.00	0.00	0.00	0.00
SO₄^2^- (mg/L)	15.21	16.13	17.19	17.79	17.4	16.61	10.22	13.18	12.53	15.24

Dissolved oxygen (O_2_); temperature (T); electrical conductivity (EC); turbidity (Turb); calcium (Ca^2+^); chloride (Cl^−^); potassium (K^+^); magnesium (Mg^+^); sodium (Na^+^); ammonium (NH^4+^); nitrite (NO_2_^−^); nitrate (NO_3_^−^); phosphate (PO_4_^3−^); sulphate (SO_4_^2−^)

Dissolved oxygen concentrations were similar across sites of both streams (Table 1). pH values in both streams varied between 7.0 and 7.7. Water temperature ranged from 9.1 to 11.2 °C in the Otterbach sites and from 8.8 to 10.5 °C in the Perlenbach sites, while it differed consistently across T1 and T2 due to seasonal effects.

### Bacterial alpha and beta diversity

**Fig. 2:**
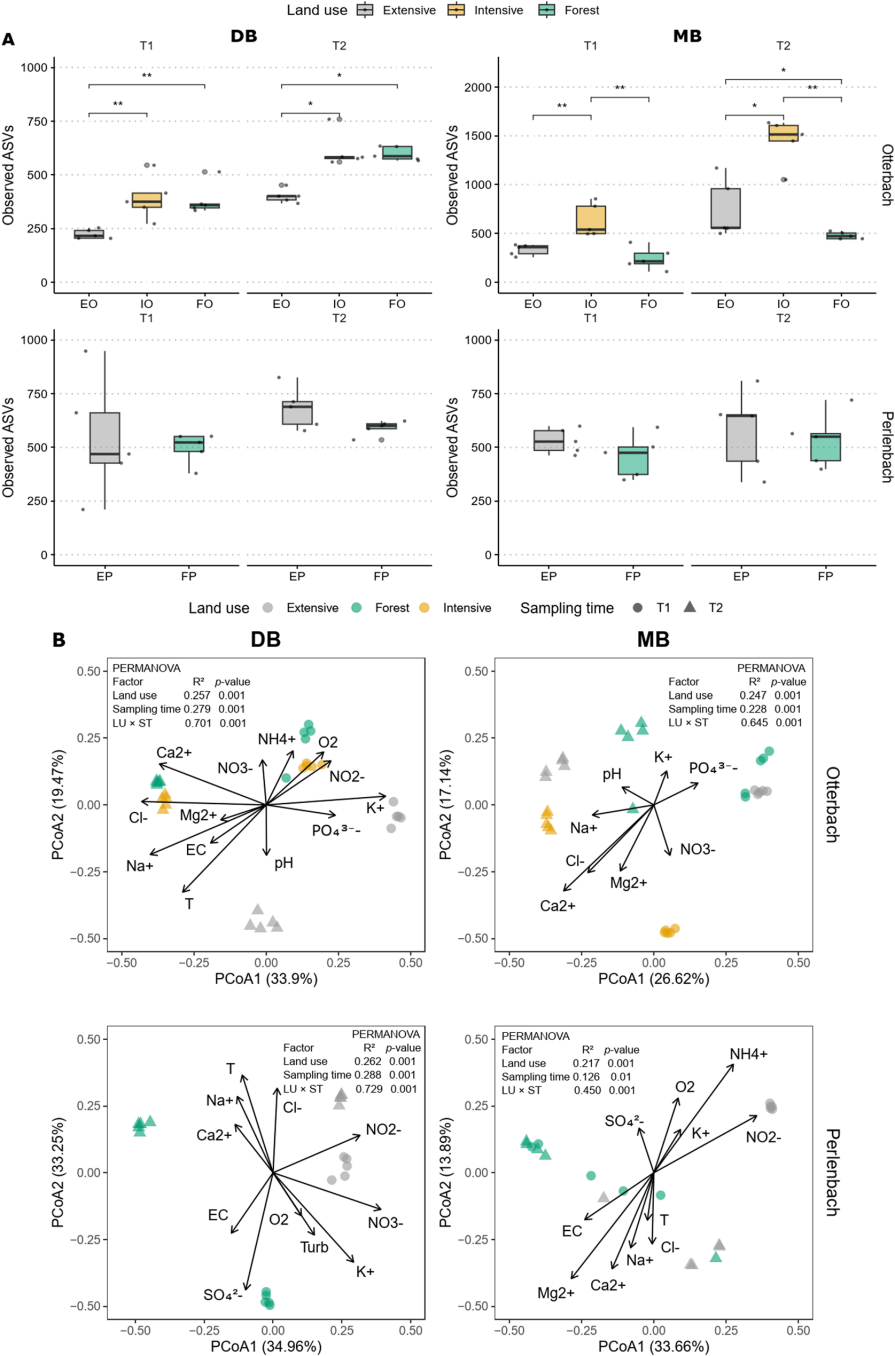
**A** Alpha diversity (observed ASVs number) in developing (DB) and mature biofilm (MB) samples from the Otterbach (EO: extensive grassland, IO: intensive farming and FO: forest) and the Perlenbach (EP: extensive grassland and FP: forest) sites. Adjusted *P* values from the Wilcoxon rank-sum test are presented in Table S2**B** Beta diversity based on the Bray Curtis distance and visualized using principal coordinates analyses, representing dissimilarity across DB and MB samples from both streams. Arrows indicate significant (permutation tests, Table S4) environmental variables (nutrients and abiotic parameters) with direction representing the gradient and length the strength of the correlation with bacterial community composition. PERMANOVA results shown in the plots indicate the significance of land use (LU) and sampling time (ST) and their interaction in explaining variation in bacterial community composition

Bacterial alpha diversity (richness), estimated as the number of observed ASVs (Fig. 2A), was highest in developing biofilms (DB) from the intensive farming site of the Otterbach stream (IO), with 502 ASVs in summer and 667 ASVs in autumn. Samples from the forest site (FO) showed intermediate richness (496 and 604 ASVs in summer and autumn, respectively), while the lowest richness values were found in the DB samples from the extensive grassland site (EO) (347 and 443 ASVs in summer and autumn, respectively). Bacterial richness differences were significant when comparing the samples from the intensive and forest site with the ones from the extensive site both in summer (*P* = 0.008) and autumn (*P* = 0.008). Additionally, richness in samples from the intensive site was significantly higher than in those from the forest site in autumn (*P* = 0.032).

A similar pattern was observed in the mature biofilm samples (MB) from the Otterbach stream; however, the number of ASVs was higher than in DB samples, mainly at the intensive farming site with 586 ASVs in summer and 1382 ASV in autumn. Interestingly, MB samples from the forest site of the Otterbach displayed the lowest bacterial richness with 302 and 534 ASVs, in summer and autumn, respectively. Significant differences in richness were only found when comparing the samples from the intensive site with those from the extensive grassland and forest site both in summer (*P* = 0.032) and in autumn (*P* = 0.016 and *P* = 0.0079 for extensive and forest, respectively).

Bacterial richness differences were not significant when comparing the samples from the forest (FP) and the extensive grassland (EP) from the Perlenbach stream (Fig. 2A) at neither of the sampling time points independent of being DB or MB samples (Table S2).

To evaluate heterogeneity, β-dispersion was assessed. The distance to the centroid increased along the stream in DB samples at the summer sampling time (Fig. S3), with the samples from FO being significantly more heterogeneous (*P* < 0.01 comped with IO and EO, respectively), while it followed the inverse pattern in DB samples at the autumn sampling time (Fig. S3), with FO samples showing the lowest heterogeneity (*P* < 0.01 compared with IO and EO, respectively). Interestingly, for MB samples IO also showed the highest heterogeneity at the summer sampling time and then the lowest in autumn (Fig. S3). In addition, the samples from the Perlenbach did not follow clear trends in terms of heterogeneity, although differences between the sites were always significant (Fig. S3).

Beta diversity, estimated using the Bray–Curtis dissimilarity index and visualized via PCoA, indicated that bacterial communities were primarily structured by adjacent land use and sampling time in both streams (Fig. 2B). This pattern was confirmed by PERMANOVA tests, which yielded significant *P* values for both variables across all sample types in both the Otterbach and Perlenbach (Table S3). Most abiotic parameters and nutrients showed significant correlations with bacterial community composition according to permutation tests (Table S4).

In the DB biofilm samples from the Otterbach (Fig. 2B), ammonium (NH₄⁺) was associated with the forest samples collected in summer, whereas oxygen (O_2_) and nitrite (NO_2_^−^) were closely associated with the summer samples from the intensive site. Potassium (K^+^) showed a strong association with the extensive site samples from the summer sampling. In autumn, calcium (Ca^2+^) and chloride (Cl^−^) were associated with samples from the forest and intensive farming site, respectively. In the MB biofilm samples from the Otterbach (Fig. 2B), the measured environmental variables were less explanatory for bacterial community composition differences between sites and sampling times, and only sodium (Na^+^) was closely associated with the autumn samples from the intensive site samples and one autumn sample from the forest site. In DB biofilm samples from the Perlenbach (Fig. 2B), nitrite and nitrate (NO_3_^−^) were associated with samples from the extensive site at the summer sampling, while sulphate (SO_4_^2−^) was explanatory of the forest site samples from the summer timepoint. Likewise, in MB samples from the Perlenbach, nitrite was correlated with the extensive site samples from the summer timepoint, while EC was correlated one samples from the forest site at the summer sampling time and another from the extensive at the autumn timepoint.

### Shifts in bacterial community composition

**Fig. 3:**
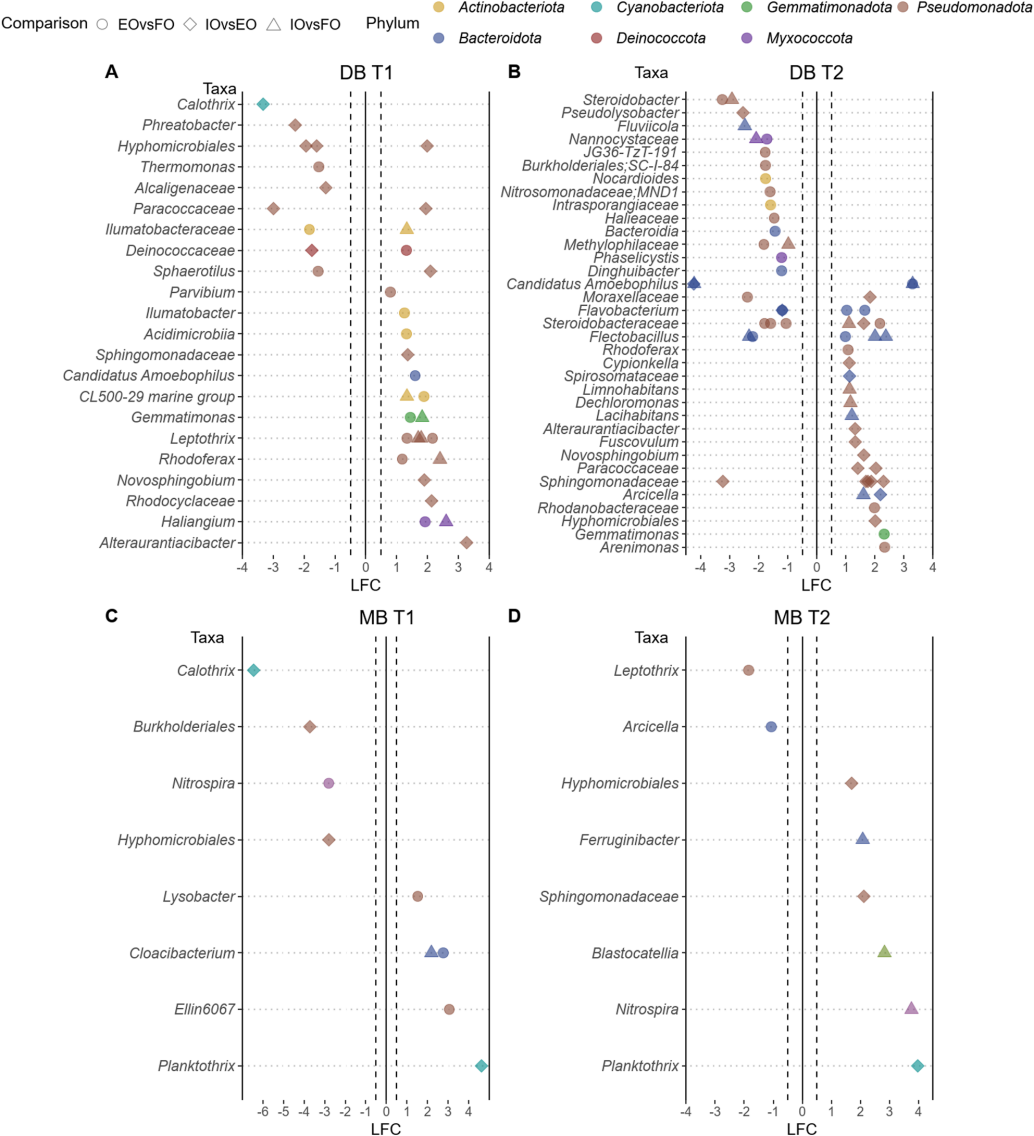
ANCOM-BC2 analyses comparing ASVs from the forest, extensive grassland and intensive farming sites of the Otterbach stream in **A** developing biofilm (DB) samples at the first (T1) and **B** second (T2) sampling times **C** mature biofilm (MB) samples at T1 and **D** at T2. Shapes indicate the comparison type: circles correspond to the comparison forest (FO) vs. extensive (EO), diamonds to intensive (IO) vs. extensive (EO) and triangles to forest (FO) vs. intensive (IO). The effect size is represented as log fold change (LFC), and colours represent the phylum to which each ASVs was assigned

The ANCOM-BC2 analyses comparing ASV abundances between the samples from the forest, extensive grassland and intensive farming sites (Fig. 3), with effect sizes estimated as log-fold change, revealed more significant differences between the Otterbach sites, with variations depending on sample type and sampling time. In contrast, only one ASV was detected when comparing DB samples from extensive and the forest Perlenbach sites at the summer sampling time (Table S5), and three ASVs for the same comparison at the autumn sampling time (Table S5). These ASVs were classified as *Alteraurantiacibacte*r, *Polymorphobacter*, *Hyphomicrobiales* and *Actinobacteria*.

In DB biofilm samples of the Otterbach stream from the summer sampling (Fig. 3A), agricultural land use resulted in 17 enriched ASVs, 6 ASVs in the intensive site and 11 ASVs in the extensive site, compared to the samples from the forest site. In contrast, only 3 ASVs were enriched in the samples from the forest site, all when compared to the extensive agricultural site, while 7 ASVs were enriched at each site when compared to the samples from the intensive site with those from the extensive (Fig. 3A). In autumn (Fig. 3B), the same number of ASVs were enriched considering the adjacent agricultural land use (17 ASVs) in DB biofilm samples, with 8 ASVs enriched at the intensive site and 9 ASVs at the extensive site. At the same time, the forest site had a stronger influence than for the summer sampling, with 24 ASVs, 5 ASVs enriched when compared to the intensive site, and 19 ASVs enriched when compared to the samples from the extensive site. 16 ASVs were enriched at the intensive site, while only 4 ASVs were differentially abundant at the extensive site when comparing the samples from those (Fig. 3B).

Fewer differentially abundant ASVs were detected in the MB biofilm samples from the Otterbach at both sampling times, as adjacent agricultural land use resulted in only 4 enriched ASVs in the summer sampling (Fig. 3C) (2 ASVs from the samples of the extensive and 2 from the samples of the intensive site compared to the forest, respectively), while only 3 ASVs, were significantly enriched in the samples from the intensive at the autumn sampling time (Fig. 3D). In addition, 3 ASVs were enriched at the extensive site, and only 1 ASV was enriched at the intensive sites when comparing the samples from those collected during the summer sampling (Fig. 3C).While at the autumn sampling, 3 ASVs were enriched at the intensive site samples when compared to those from the extensive site (Fig. 3D).

*Pseudomonadota* with 14 ASVs and *Bacteroidota* with 13 ASVs were the most enriched phyla by adjacent agricultural land use (Fig. 3A–D). Moreover, several ASVs were enriched both in the samples from the intensive and the extensive site when compared to the forest. Of those, CL500-29 marine group, *Gemmatimonas*, *Leptothrix*, *Rhodoferax* and *Haliangium* were enriched in the DB biofilm samples from the summer sampling, while *Steroidobacteraceae, Candidatus Amoebophilus* and *Flectobacillus* in the DB samples from the autumn sampling, and *Cloacibacterium* in the MB biofilm samples from the summer timepoint.

### Bacterial community assembly analyses

**Fig. 4:**
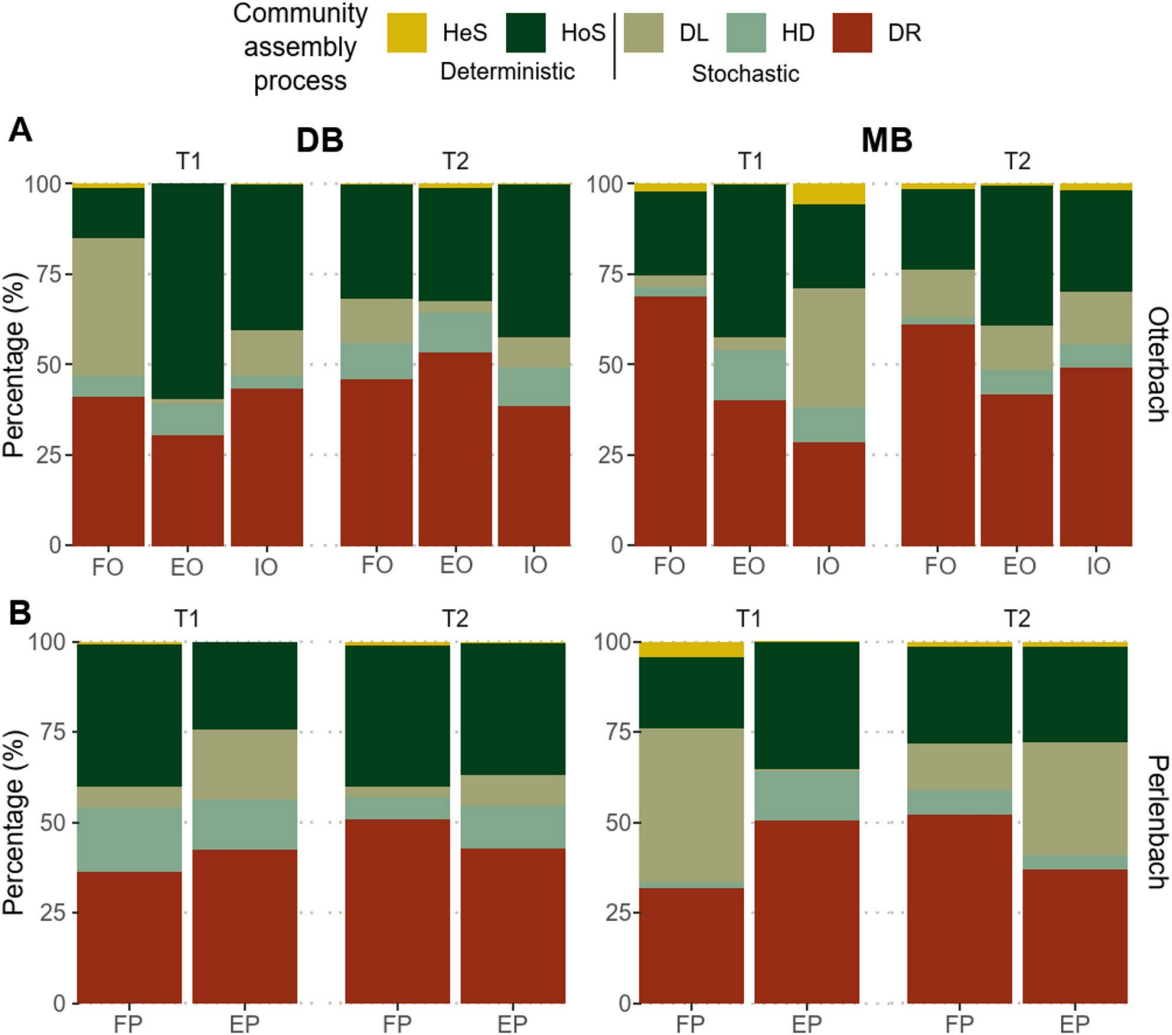
Variation in the relative importance of bacterial community assembly processes estimated with iCAMP in **A** Developing (DB) and mature biofilm (MB) samples from the three sites of the Otterbach at the summer (T1) and autumn (T2) timepoints and **B** DB and MB samples from the two sites of the Perlenbach at T1 and T2. Assembly processes include heterogeneous selection (HeS), homogeneous selection (HoS), dispersal limitation (DL), homogenizing dispersal (HD) and drift (DR). Percentages are displayed in Table S7

The findings of the iCAMP analysis indicated that homogeneous selection, drift and dispersal limitation were the major drivers of bacterial community assembly across biofilm samples from both streams, as seen in Fig. 4A and B, which represent the variation in the relative importance of bacterial community assembly processes in DB and MB samples from the Otterbach and Perlenbach. In particular, homogeneous selection was the process with the highest relative abundance in the Otterbach DB (59.6%) and MB (42.4%) samples from the extensive site at the first sampling time, and of the DB biofilm samples from the intensive agricultural site at the autumn sampling time (42.3%). Contrarily, drift displayed special relevance in the MB biofilm samples from the forest site at both the summer (68.6%) and autumn sampling time (61.0%). When examining which bins were driving the main bacterial community assembly processes at each sample type, site and sampling time in the Otterbach stream, we found the bacterial taxa described in Table S6. In addition, their taxonomic classification was retrieved, along with their contribution to the relative importance of the assembly process.

Drift was the assembly process with the highest relative importance (42.9% and 42.7%, respectively) in the majority of DB and MB biofilm samples from the Perlenbach (Fig. 4B), while homogeneous selection and dispersal limitation displayed the highest relative importance in the DB forest samples of the summer sampling time (39.6%) and the MB biofilm samples from the forest site of the same sampling time (42.4%), respectively.

### Bacterial co-occurrence networks analysis

**Fig. 5:**
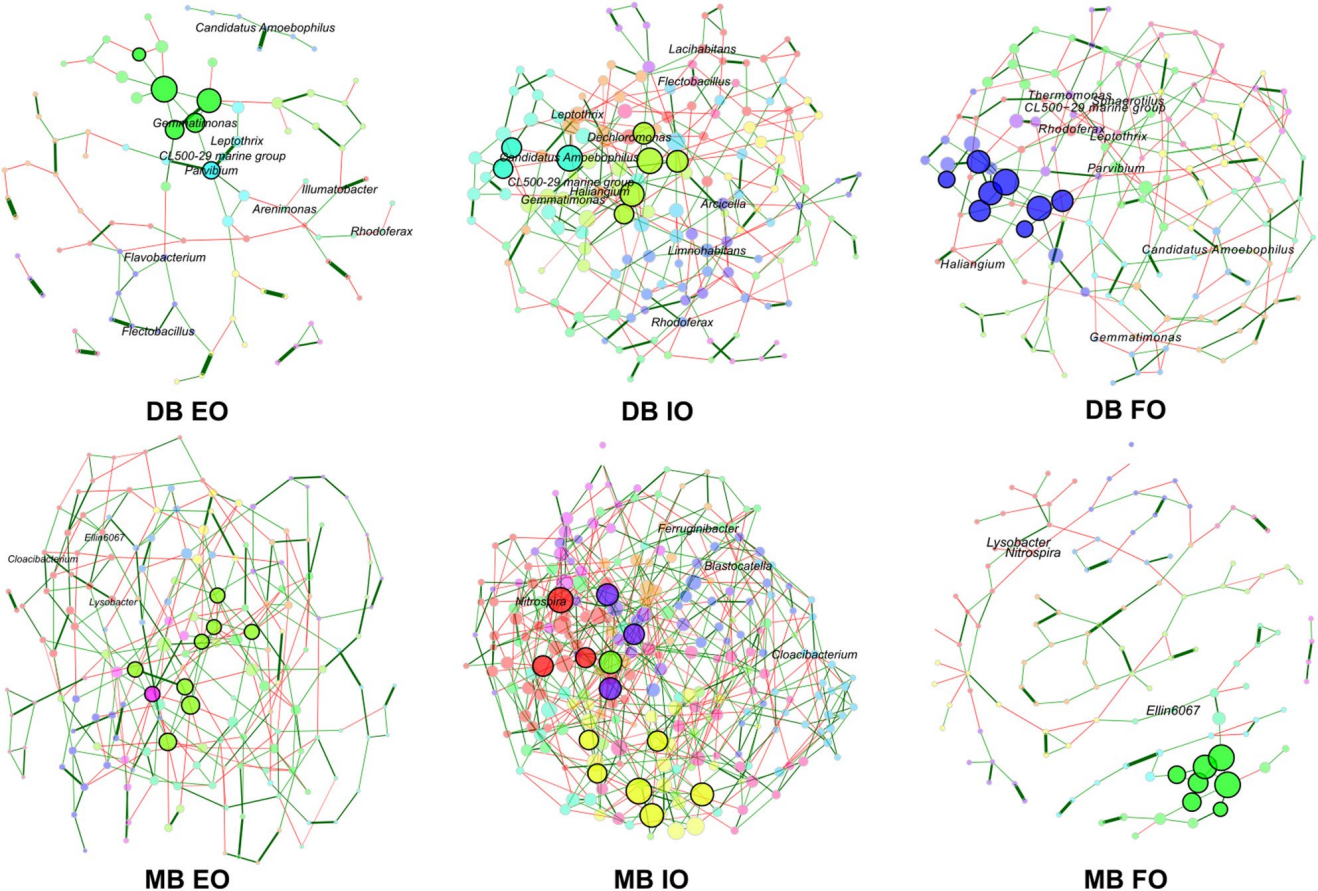
Co-occurrence networks of the most abundant bacterial taxa in developing (DB) and mature biofilms (MB) from the Otterbach sites: extensive grassland (EO), intensive farming (IO) and forest (FO). Despite significant β-diversity differences between sampling times, the networks were constructed combining samples from the two sampling times (N = 10), following established practices in microbial co-occurrence network analysis to reliably retrieve robust co-occurrence patterns rather than time specific dynamics. Only nodes corresponding to bacterial taxa highlighted in the differential abundance and iCAMP analyses are labelled. Nodes are coloured according to cluster, groups of strongly connected taxa. Edge colour and thickness indicate the sign and strength of the association, respectively. Positive associations are displayed in green and negative in red, while thicker edges represent stronger correlations

To identify the role of the taxa consistently responding to adjacent land use over time in the biofilm bacterial communities, we performed co-occurrence network analyses. The co-occurrence networks revealed clear differences in network topology and community structure (Fig. 5), including a substantial increase in nodes number from DB to MB biofilms in the samples from both the extensive (97 and 168, respectively) and the intensive agricultural (157 and 246, respectively) site of the Otterbach. In contrast, this increase was not observed in the samples from the forest site of the Otterbach (155 and 127 in DB and MB, respectively). The networks from the DB biofilm samples of the intensive site, as well as from the MB biofilm samples of the extensive and intensive agricultural sites consisted of a single component, as all the nodes were connected directly or indirectly (Table S8). In contrast, the network from DB biofilm from the extensive agricultural site of the Otterbach was more fragmented, consisting of 13 components (Table S8) and displaying the highest modularity (0.774, Table S8). Moreover, the networks from both DB and MB biofilm samples of the intensive farming site displayed the lowest modularity values (Table S8).

The networks of the Perlenbach biofilm DB samples (Fig. S4 and Table S8) displayed the same trends as the DB biofilm samples of the Otterbach, in particular the ones from the extensive agricultural site, which displayed a high number of components. Also, MB biofilm samples from both Otterbach and Perlenbach showed similarities, especially in the networks of the forest sites which had a higher number of components and fewer nodes (Table S8).

Several bacterial taxa previously identified as enriched under agricultural influence and driving the increase in relative importance of deterministic processes (e.g., *Arenimonas*, *Arcicella*, *Cloacibacterium*, CL500-29 marine group, *Dechloromonas*, *Ferruginibacter, Flectobacillus*, *Leptothrix*, *Lysobacter*, *Nitrospira* or *Rhodoferax*) displayed high connectivity in the networks (Fig. 5), some of which were even part of the same module, such as *Cloacibacterium* and *Lysobacter, Leptothrix,* CL500-29 marine group and *Parvibium,* or *Dechloromonas* and *Lacihabitans*, while some enriched taxa played a key role in the co-occurrence networks as main module hubs such as *Candidatus Amoebophilus*, *Gemmatimonas* and *Parvibium;* Table S9).

## Discussion

In this study, we demonstrated that agricultural land use, as extensive grassland and intensive farming, differentially affects bacterial diversity, community composition, and assembly of adjacent freshwater microbial biofilms. These effects varied with the stage of biofilm development and were distinct from those observed in biofilms from the forest site.

Notably, while the stream exposed to less anthropogenic impact (Perlenbach), as indicated by the lower nutrient loads, displayed some differences in bacterial community composition between the forest and the extensive grassland site, clear effects on bacterial community composition of developing and mature biofilms were only observed in the stream with higher anthropogenic influence (Otterbach), and increased nutrient concentrations. These changes were primarily driven by an increase in the relative abundance of bacterial taxa that not only contributed to the rise in deterministic processes in bacterial community assembly but also held high centrality and connectivity within bacterial co-occurrence networks. The observed effects were consistent across the summer and autumn samplings.

### Factors explaining the differences between the Otterbach and Perlenbach stream

Previous studies have highlighted the importance of nutrient concentrations when assessing differences in water quality between streams [52]. In this case study, nutrient concentrations were substantially lower in the Perlenbach sites, consistent with the reduced human activity in the area, including the absence of fertilization and settlements. In contrast, several nutrients were found at higher concentrations in the Otterbach sites, in particular, nitrate and potassium, which are typical fertilizer components [53] that get commonly flushed into watersheds from adjacent agricultural sites during storm events [54], and magnesium or calcium, which can end up in watersheds due to liming in croplands [55].

Another factor that could potentially explain the lack of significant differences between the Perlenbach sites is their close proximity (113 m), which would contribute to the homogenization of bacterial communities through dispersion as shown in a simplified synthetic metacommunity [56]. While the sites of the Otterbach were several kilometres away from each other (4.01 km from EO to IO and 3.5 km from IO to FO).

Drift and dispersal limitation played a key role in bacterial community assembly in the Perlenbach stream samples, which goes in agreement with the lack of stressors and disturbance in this stream, as environmental stressors normally increase the relative importance of deterministic processes [57].

### Developing biofilms showed increased vulnerability to adjacent land use

Stream biofilms can be influenced by physical, chemical and biological stressors [58]. In our study, developing biofilms proved to be more sensitive to adjacent land use, as they displayed stronger responses across analyses in the Otterbach stream, which highlights their potential as bioindicators, and suggest a shift into an alternative state as shown in early-stage biocrusts [59]. This included notable shifts in bacterial richness (Fig. 2A), a stronger influence of physicochemical variables (Fig. 2B) and a higher number of enriched ASVs in comparison with MB biofilm samples (Fig. 3). Interestingly, DB biofilm samples from the site adjacent to the extensive grassland land use exhibited the lowest bacterial richness and the highest share of deterministic processes. This suggests the influence of environmental filtering and priority effects [60], which limited colonization to a few well-adapted taxa, leading to low alpha diversity and a high number of components and modularity of the bacterial co-occurrence network (Fig. 5). This land use type has previously been linked to perturbations in nearby freshwater biofilms [61], and its proximity to a village may have intensified stressors, such as oxidative stress, or sewage inputs [62].

In contrast, the DB samples from the adjacent intensive agricultural site exhibited the highest bacterial richness values, likely due to more nutrient-rich conditions, including higher availability of labile dissolved organic carbon (DOC) (Fig. S5), supporting a broader range of colonizers in the biofilms. This effect became more pronounced in the autumn sampling time, when DB samples exhibited an even higher bacterial richness, likely due to the presence of more labile compounds and more diverse niches for bacteria, while the bacterial communities of the forest site may have experienced niche monopolization by highly specialized bacteria and fungi capable of effectively degrading inputs from fallen leaves [63, 64], such as *Comamonadaceae*. Although precipitation levels were lower in autumn (Fig. S6), the mentioned input of leave material would have still provided a consistent source of available nutrients, which is also evidenced in the higher turbidity in all sites at this sampling time (Table 1).

The phylum with the most enriched ASVs due to agricultural land use in DB samples was *Pseudomonadota*. This is in line with previous research, which identified this phylum as highly responsive to nutrient pollution, in particular to total nitrogen and total phosphorus from agriculture and urbanization [65]. Furthermore, several taxa were enriched in both the extensive and intensive site samples when compared to the ones from the forest site, such as CL-500-29 marine group, *Gemmatimonas*, *Leptothrix*, *Rhodoferax* and *Haliangium* at the summer sampling time, and *Steroidobacteraceae*, *Flectobacillus* and *Candidatus Amoebophilus* at the autumn sampling time. Determining whether these taxa originate from the adjacent agricultural land use is challenging, as both water and soil have been proven to contribute equally as a source of bacteria to biofilms [66]. A clear example of this is *Gemmatimonas*, which has been identified performing N_2_O reduction in agricultural soils [67] but can also perform photosynthesis in freshwater ecosystems [68], being potentially involved in both.

In addition, we found a consistent overlap between the enriched bacteria and the taxa behind the rise in relative importance of deterministic processes in the iCAMP analysis. This pattern supports previous research conducted in agricultural soils, which showed how environmental filtering, via nutrient input, redox dynamics, and abiotic stress, amplifies deterministic processes under agricultural land use [69]. Some of these taxa were *Gemmatimonas* and *Leptothrix,* which has been previously described as typical from wastewater treatment plants and iron-rich freshwater [70] or *Rhodoferax*, which can carry out denitrification and was previously detected in wastewater and polluted water [71]. Interestingly, the increased presence of wastewater-associated bacteria in stream biofilms has been associated to a higher tolerance towards micropollutants [72].

### Mature biofilms remained more resilient to the influence of adjacent land use

In contrast to developing biofilm samples, mature biofilms were more resistant to the effects of adjacent land use, especially in the Otterbach stream, where only the samples from the intensive site underwent a strong increase in richness. This finding aligns with previous research [73] which showed that stream biofilm assemblages developed according to successional stages, characterized by an increase in complexity and richness, which could potentially enhance their resilience to environmental disturbances. Interestingly, the extensive and forest samples from both Otterbach and Perlenbach exhibited similar richness and variability. This increase in richness at the intensive site may be attributed to higher nutrient availability [74], especially the input of labile DOC. Additionally, autumn leaf litter likely enhanced substrate diversity and further contributed to the rise in bacterial richness (Fig. 2A). Moreover, the increase in bacterial co-occurrence network complexity from DB to MB biofilm samples resembles the tendency described in forest soil microbial communities along successional stages [75].

Additional evidence of the greater stability of mature biofilms is provided by the lower number of environmental factors significantly affecting the Otterbach MB bacterial community composition compared to DB, including fewer significant physicochemical parameters and shorter vector lengths (Fig. 2B). Further proof of this increased resilience of mature biofilms were the fewer shifting ASVs in the differential abundance analyses when comparing the forest site samples against those from the extensive and the intensive site (Fig. 3C and D) and the more stable co-occurrence networks (Fig. 5), with fewer modularity and no more than three components (Table S6). Interestingly, the only bacterial taxa whose ASVs resulted differentially abundant under intensive and extensive land use in MB samples was *Cloacibacterium* (Fig. 3C), which is commonly found in wastewater and has been described in studies from wastewater and urban polluted streams [76] and can perform denitrification [77].

## Conclusions

Our study demonstrates how adjacent agricultural land use differentially shaped bacterial richness, bacterial community composition and bacterial community assembly in developing and mature biofilms driven by an increase in nutrient concentrations and the proliferation of key responding bacterial taxa in the stream undergoing higher anthropogenic pressure. These differences between streams were linked to increased nutrient concentrations from the anthropogenic influence and showcased the higher vulnerability of developing biofilms, which can shift towards alternative assembly trajectories, with potential repercussions for nutrient cycling and microbial interactions in streams. Such land use-driven restructuring of biofilm communities may propagate through freshwater food webs, potentially altering ecological roles and resilience in human-impacted catchments.

As this was an observational field study, we cannot determine whether the enriched bacteria thrived due to nutrient shifts from the agricultural land use or there was a biotic transport of bacteria that effectively colonized the biofilms. Moreover, fungi and algae may have also played a role in explaining the observed differences through their interactions with bacteria. To more precisely classify and to obtain further insights on the ecological function of the key taxa enriched by adjacent agricultural land use, a metagenomics-based functional profiling would be required.

## Supplementary Information

Below is the link to the electronic supplementary material.

Supplementary Material 1

## Data Availability

The 16S rRNA gene amplicon sequencing dataset is available in the Sequence Read Archive (SRA) of NCBI and can be accessed via the accession ID: PRJNA1271419. All scripts, metadata and physicochemical properties dataset of the study are available at Zenodo: 10.5281/zenodo.16813660.
