# Supplementary material for: Agricultural land use shapes short and long‑term bacterial diversity, community structure, and assembly in biofilms of adjacent streams
Source: Environ Microbiome. 2025 Dec 20;21:17. doi: 10.1186/s40793-025-00837-9 (PMC12837902; doi:10.1186/s40793-025-00837-9)

**Supplementary Material**

**Agricultural land use shapes short and long‑term bacterial diversity, community structure, and assembly in biofilms of adjacent streams**

Rubén Martínez-Cuesta^12*^; Rebecca Hoess^3^; Sebastian Floßmann^4^; Juergen Geist^3^; Michael Dannenmann^4^; Michael Schloter^12^; Stefanie Schulz^2^

**Supplementary Tables**

**Table S1** Overview of the number of reads at each step of the DADA2 pipeline.

| SampleID | Sample type | Site | Sampling time | Stream | input | filtered | denoisedF | denoisedR | merged | nonchim |
| --- | --- | --- | --- | --- | --- | --- | --- | --- | --- | --- |
| RM-100 | MB | IO | T1 | Otterbach | 47487 | 31976 | 28091 | 29434 | 22512 | 22025 |
| RM-101 | MB | IO | T1 | Otterbach | 65027 | 44700 | 39715 | 41266 | 31123 | 30527 |
| RM-102 | MB | IO | T1 | Otterbach | 78461 | 53095 | 47038 | 49050 | 36651 | 35845 |
| RM-103 | MB | IO | T1 | Otterbach | 44049 | 28887 | 24865 | 26277 | 18864 | 18553 |
| RM-104 | MB | IO | T1 | Otterbach | 45024 | 29910 | 26315 | 27682 | 21031 | 20515 |
| RM-105 | extraction_negative | negative | T1 | negative | 221 | 129 | 91 | 105 | 71 | 68 |
| RM-106 | pcr_negative | negative | T2 | negative | 510 | 333 | 299 | 272 | 250 | 236 |
| RM-182 | DB | FO | T2 | Perlenbach | 45395 | 31595 | 28545 | 29267 | 21931 | 21828 |
| RM-183 | DB | FO | T2 | Perlenbach | 55231 | 38002 | 34393 | 35630 | 26880 | 26720 |
| RM-184 | DB | FO | T2 | Perlenbach | 48679 | 33766 | 30571 | 31468 | 23495 | 23382 |
| RM-185 | DB | FO | T2 | Perlenbach | 44926 | 30794 | 27761 | 28491 | 20928 | 20851 |
| RM-186 | DB | FO | T2 | Perlenbach | 52948 | 36005 | 32657 | 33534 | 25106 | 24899 |
| RM-187 | DB | EP | T2 | Perlenbach | 48382 | 31783 | 28058 | 29077 | 19863 | 19720 |
| RM-188 | DB | EP | T2 | Perlenbach | 39290 | 26148 | 22843 | 23751 | 16066 | 15955 |
| RM-189 | DB | EP | T2 | Perlenbach | 49591 | 33594 | 29838 | 30842 | 20304 | 20136 |
| RM-190 | DB | EP | T2 | Perlenbach | 57052 | 39090 | 34941 | 36110 | 25234 | 24964 |
| RM-191 | DB | EP | T2 | Perlenbach | 40515 | 26895 | 23607 | 24472 | 16257 | 16144 |
| RM-192 | DB | FO | T2 | Otterbach | 44462 | 30080 | 26914 | 27461 | 19538 | 19466 |
| RM-193 | DB | FO | T2 | Otterbach | 47089 | 31791 | 28222 | 29352 | 20705 | 20625 |
| RM-194 | DB | FO | T2 | Otterbach | 51428 | 34751 | 31158 | 32166 | 23123 | 23030 |
| RM-195 | DB | FO | T2 | Otterbach | 49445 | 32342 | 28948 | 29850 | 22104 | 21976 |
| RM-196 | DB | FO | T2 | Otterbach | 47036 | 32211 | 28817 | 29740 | 21502 | 21428 |
| RM-197 | DB | EO | T2 | Otterbach | 53248 | 36256 | 34034 | 34871 | 29772 | 28417 |
| RM-198 | DB | EO | T2 | Otterbach | 56131 | 38768 | 36908 | 37316 | 32488 | 30914 |
| RM-199 | DB | EO | T2 | Otterbach | 60562 | 41319 | 38691 | 39463 | 33085 | 31755 |
| RM-200 | DB | EO | T2 | Otterbach | 46712 | 31767 | 29662 | 30236 | 25054 | 24179 |
| RM-201 | DB | EO | T2 | Otterbach | 56776 | 38353 | 35857 | 36578 | 30869 | 29393 |
| RM-202 | DB | IO | T2 | Otterbach | 46293 | 31503 | 28035 | 28834 | 21772 | 21645 |
| RM-203 | DB | IO | T2 | Otterbach | 54233 | 35291 | 31513 | 32731 | 24378 | 24211 |
| RM-204 | DB | IO | T2 | Otterbach | 54711 | 37307 | 33833 | 34856 | 26451 | 26275 |
| RM-205 | DB | IO | T2 | Otterbach | 57087 | 38781 | 35090 | 36141 | 26549 | 26460 |
| RM-206 | DB | IO | T2 | Otterbach | 67537 | 46300 | 42212 | 43454 | 33366 | 33066 |
| RM-207 | MB | FO | T2 | Otterbach | 60258 | 40968 | 37617 | 38766 | 30002 | 29678 |
| RM-208 | MB | FO | T2 | Otterbach | 56470 | 38534 | 35581 | 36389 | 29989 | 29782 |
| RM-209 | MB | FO | T2 | Otterbach | 59385 | 40266 | 37533 | 38275 | 31756 | 31585 |
| RM-210 | MB | FO | T2 | Otterbach | 59615 | 40492 | 38242 | 38811 | 33610 | 33379 |
| RM-211 | MB | FO | T2 | Perlenbach | 52302 | 34856 | 32901 | 33464 | 29115 | 28925 |
| RM-212 | MB | EP | T2 | Perlenbach | 57720 | 38070 | 34912 | 35757 | 28037 | 27569 |
| RM-213 | MB | EP | T2 | Perlenbach | 60322 | 40283 | 35747 | 37232 | 26105 | 25840 |
| RM-214 | extraction_negative | negative | T2 | negative | 50373 | 34479 | 33461 | 33484 | 30056 | 29765 |
| RM-215 | extraction_negative | negative | T2 | negative | 1688 | 1134 | 1084 | 1077 | 956 | 904 |
| RM-216 | extraction_negative | negative | T2 | negative | 2306 | 1554 | 1442 | 1405 | 1234 | 1218 |
| RM-217 | pcr_negative | negative | T2 | negative | 255 | 169 | 155 | 153 | 151 | 149 |
| RM-218 | MB | EP | T2 | Perlenbach | 34387 | 22948 | 20573 | 20988 | 15125 | 14978 |
| RM-219 | MB | EP | T2 | Perlenbach | 45613 | 31053 | 27067 | 28122 | 18995 | 18815 |
| RM-220 | MB | EP | T2 | Perlenbach | 45408 | 31527 | 29017 | 29577 | 23192 | 23012 |
| RM-221 | MB | FO | T2 | Otterbach | 43977 | 29563 | 26355 | 27209 | 20727 | 20598 |
| RM-222 | MB | FO | T2 | Otterbach | 42953 | 26967 | 23980 | 24762 | 18191 | 18154 |
| RM-223 | MB | FO | T2 | Otterbach | 42869 | 28490 | 25324 | 26353 | 19659 | 19573 |
| RM-224 | MB | FO | T2 | Otterbach | 37642 | 25585 | 23220 | 23884 | 18738 | 18576 |
| RM-225 | MB | FO | T2 | Otterbach | 39629 | 26624 | 23717 | 24527 | 18198 | 18138 |
| RM-226 | MB | EO | T2 | Otterbach | 44431 | 30252 | 26095 | 27485 | 18805 | 18706 |
| RM-227 | MB | EO | T2 | Otterbach | 42724 | 28486 | 24688 | 25860 | 17339 | 17244 |
| RM-228 | MB | EO | T2 | Otterbach | 45912 | 30584 | 26745 | 27856 | 18445 | 18300 |
| RM-229 | MB | EO | T2 | Otterbach | 102136 | 62003 | 55746 | 57937 | 41755 | 41404 |
| RM-230 | MB | EO | T2 | Otterbach | 118970 | 79597 | 73048 | 75119 | 57004 | 56395 |
| RM-231 | MB | IO | T2 | Otterbach | 90061 | 61612 | 56234 | 57819 | 44503 | 44116 |
| RM-232 | MB | IO | T2 | Otterbach | 138110 | 94297 | 87488 | 89494 | 71486 | 70351 |
| RM-233 | MB | IO | T2 | Otterbach | 152798 | 101829 | 94940 | 97008 | 80163 | 79133 |
| RM-234 | MB | IO | T2 | Otterbach | 138188 | 93945 | 87488 | 89541 | 72476 | 71451 |
| RM-235 | MB | IO | T2 | Otterbach | 166848 | 111993 | 104295 | 106675 | 85501 | 84355 |
| RM-236 | extraction_negative | negative | T2 | negative | 3206 | 2088 | 1802 | 1793 | 1406 | 1331 |
| RM-237 | pcr_negative | negative | T2 | negative | 390 | 228 | 187 | 178 | 178 | 167 |
| RM-51 | DB | FO | T1 | Perlenbach | 102561 | 67981 | 65748 | 66427 | 60721 | 59947 |
| RM-52 | DB | FO | T1 | Perlenbach | 88322 | 60066 | 58638 | 58910 | 54177 | 53618 |
| RM-53 | DB | FO | T1 | Perlenbach | 92470 | 63332 | 60683 | 61287 | 54342 | 53776 |
| RM-54 | DB | FO | T1 | Perlenbach | 102377 | 69172 | 66456 | 67367 | 59744 | 58835 |
| RM-55 | DB | FO | T1 | Perlenbach | 89729 | 59537 | 56777 | 57624 | 50658 | 50086 |
| RM-56 | DB | EP | T1 | Perlenbach | 64363 | 43332 | 39700 | 40832 | 32187 | 32026 |
| RM-57 | DB | EP | T1 | Perlenbach | 114729 | 78078 | 72107 | 74144 | 59722 | 58848 |
| RM-58 | DB | EP | T1 | Perlenbach | 48390 | 32434 | 29567 | 30287 | 24391 | 24272 |
| RM-59 | DB | EP | T1 | Perlenbach | 48993 | 33208 | 30650 | 31378 | 25636 | 25432 |
| RM-60 | DB | EP | T1 | Perlenbach | 22237 | 15136 | 13565 | 13975 | 10555 | 10468 |
| RM-61 | DB | FO | T1 | Otterbach | 45194 | 31248 | 28091 | 29063 | 21046 | 20635 |
| RM-62 | DB | FO | T1 | Otterbach | 38491 | 26438 | 22352 | 23929 | 15563 | 15310 |
| RM-63 | DB | FO | T1 | Otterbach | 32800 | 22217 | 19293 | 20289 | 14443 | 14116 |
| RM-64 | DB | FO | T1 | Otterbach | 38552 | 26180 | 23279 | 23976 | 18170 | 17884 |
| RM-65 | DB | FO | T1 | Otterbach | 41127 | 28039 | 24915 | 25942 | 19781 | 19497 |
| RM-66 | DB | EO | T1 | Otterbach | 43360 | 27820 | 25968 | 26636 | 23081 | 22537 |
| RM-67 | DB | EO | T1 | Otterbach | 42628 | 28993 | 27432 | 27978 | 24562 | 23863 |
| RM-68 | DB | EO | T1 | Otterbach | 33813 | 22644 | 21106 | 21443 | 18295 | 17771 |
| RM-69 | DB | EO | T1 | Otterbach | 39260 | 26686 | 25378 | 25757 | 22954 | 22419 |
| RM-70 | DB | EO | T1 | Otterbach | 33371 | 22433 | 20754 | 21263 | 17976 | 17784 |
| RM-71 | DB | IO | T1 | Otterbach | 32890 | 21889 | 19694 | 20359 | 15698 | 15547 |
| RM-72 | DB | IO | T1 | Otterbach | 47464 | 31847 | 29000 | 29609 | 23322 | 23136 |
| RM-73 | DB | IO | T1 | Otterbach | 42587 | 28676 | 26020 | 26852 | 20872 | 20704 |
| RM-74 | DB | IO | T1 | Otterbach | 33920 | 22213 | 19462 | 20315 | 14947 | 14859 |
| RM-75 | DB | IO | T1 | Otterbach | 74972 | 50880 | 46842 | 48127 | 38604 | 38063 |
| RM-76 | MB | FO | T1 | Perlenbach | 48258 | 32477 | 30142 | 30837 | 24677 | 23926 |
| RM-77 | MB | FO | T1 | Perlenbach | 69432 | 48005 | 45354 | 46144 | 38337 | 37633 |
| RM-78 | MB | FO | T1 | Perlenbach | 80567 | 55382 | 51805 | 52986 | 45607 | 44727 |
| RM-79 | MB | FO | T1 | Perlenbach | 50599 | 34540 | 32653 | 33174 | 28891 | 28574 |
| RM-80 | MB | FO | T1 | Perlenbach | 52999 | 36004 | 33237 | 34219 | 28124 | 27802 |
| RM-81 | MB | EP | T1 | Perlenbach | 49663 | 33641 | 30516 | 31509 | 23129 | 22894 |
| RM-82 | MB | EP | T1 | Perlenbach | 44395 | 27634 | 24813 | 25863 | 18384 | 18124 |
| RM-83 | MB | EP | T1 | Perlenbach | 48258 | 33324 | 30383 | 31329 | 23202 | 23019 |
| RM-84 | MB | EP | T1 | Perlenbach | 43390 | 27867 | 25139 | 26073 | 19385 | 19180 |
| RM-85 | MB | EP | T1 | Perlenbach | 46088 | 31886 | 28916 | 29915 | 22189 | 21950 |
| RM-86 | MB | FO | T1 | Otterbach | 57337 | 38909 | 37334 | 37870 | 34807 | 34164 |

**Table S2** *P* adjusted values of the Wilcoxon rank-sum test to address differences in bacterial alpha diversity in developing (DB) and mature biofilm (MB) samples between sites at the summer (T1) and autumn (T2) sampling times.

| **Comparison** | **Sampling time** | **Biofilm** | **Stream** | ***P*-adj** |
| --- | --- | --- | --- | --- |
| FO vs EO | T1 | DB | Otterbach | 0.008 |
| FO vs IO | T1 | DB | Otterbach | 1 |
| EO vs IO | T1 | DB | Otterbach | 0.008 |
| FO vs EO | T2 | DB | Otterbach | 0.008 |
| FO vs IO | T2 | DB | Otterbach | 0.032 |
| EO vs IO | T2 | DB | Otterbach | 0.008 |
| FO vs EO | T1 | MB | Otterbach | 0.310 |
| FO vs IO | T1 | MB | Otterbach | 0.032 |
| EO vs IO | T1 | MB | Otterbach | 0.032 |
| FO vs EO | T2 | MB | Otterbach | 0.170 |
| FO vs IO | T2 | MB | Otterbach | 0.008 |
| EO vs IO | T2 | MB | Otterbach | 0.016 |
| FP vs EP | T1 | DB | Perlenbach | 0.750 |
| FP vs EP | T2 | DB | Perlenbach | 1 |
| FP vs EP | T1 | MB | Perlenbach | 0.550 |
| FP vs EP | T2 | MB | Perlenbach | 0.840 |

**Table S3** Results of the PERMANOVA analysis addressing the effect of land use, sampling time and their intercept on beta diversity (estimated with the Bray-Curtis index).

| **Otterbach** | | | | | | | | | | |
| --- | --- | --- | --- | --- | --- | --- | --- | --- | --- | --- |
| **DB** | | | | **MB** | | | | | | |
| PERMANOVA | F | R2 | *P*-value | PERMANOVA | | F | | R2 | | *P*-value |
| Land use | 4.668 | 0.257 | 0.001 *** | Land use | | 4.668 | | 0.257 | | 0.001 *** |
| Sampling time | 10.868 | 0.279 | 0.001 *** | Sampling time | | 10.868 | | 0.279 | | 0.001 *** |
| Land use x Sampling time | 6.018 | 0.701 | 0.001*** | Land use x Sampling time | | 10.213 | | 0.645 | | 0.001*** |
| **Perlenbach** | | | | | | | | | | |
| **DB** | | | | **MB** | | | | | | |
| PERMANOVA | F | R2 | *P*-value | PERMANOVA | F | | R2 | | *P*-value | |
| Land use | 6.3961 | 0.262 | 0.001 *** | Land use | 5.0055 | | 0.217 | | 0.001 *** | |
| Sampling time | 7.275 | 0.288 | 0.001 *** | Sampling time | 2.6076 | | 0.126 | | 0.017 * | |
| Land use x Sampling time | 3.847 | 0.729 | 0.001*** | Land use x Sampling time | 6.594 | | 0.450 | | 0.001*** | |

**Table S4** P-adj values, strength of the effect as length of the vector and vector orientation from the permutation tests included in Fig. 2B assessing the correlation between the measured physicochemical parameters and the variation in bacterial community composition.

| **Nutrients** | **Otterbach** | | | | | | **Perlenbach** | | | | | |
| --- | --- | --- | --- | --- | --- | --- | --- | --- | --- | --- | --- | --- |
|  | **DB** | | | **MB** | | | **DB** | | | **MB** | | |
|  | ***P*-adj** | **Length** | ***Vector orientation (°)*** | ***P*-adj** | **Length** | ***Vector orientation* (°)** | ***P*-adj** | **Length** | ***Vector orientation* (°)** | ***P*-adj** | **Length** | ***Vector orientation (°)*** |
| **Ca^2+^** | 0.001 | 0.799 | 157.446 | 0.001 | 0.896 | -134.094 | 0.009 | 0.455 | 127.345 | 0.001 | 0.775 | -111.902 |
| **Cl^-^** | 0.001 | 0.862 | 178.300 | 0.001 | 0.682 | -131.974 | 0.001 | 0.633 | 87.265 | 0.005 | 0.533 | -91.261 |
| **K^+^** | 0.001 | 0.413 | 4.509 | 0.32 | 0.269 | 70.706 | 0.001 | 0.889 | -48.852 | 0.038 | 0.372 | 60.363 |
| **Mg^2+^** | 0.006 | 0.333 | -160.413 | 0.001 | 0.546 | -115.117 | 0.166 | 0.276 | -172.757 | 0.001 | 0.977 | -125.769 |
| **Na^+^** | 0.001 | 0.883 | -155.128 | 0.003 | 0.432 | -169.887 | 0.001 | 0.627 | 114.488 | 0.006 | 0.585 | -105.901 |
| **NH_4_^+^** | 0.011 | 0.445 | 65.391 | 0.887 | 0.001 | 166.214 | 0.186 | 0.244 | 4.573 | 0.001 | 0.981 | 55.907 |
| **NO_2_^-^** | 0.001 | 0.557 | 36.373 | 0.407 | 0.015 | 146.104 | 0.002 | 0.691 | 23.977 | 0.001 | 0.830 | 30.970 |
| **NO_3_^-^** | 0.05 | 0.338 | 94.429 | 0.01 | 0.394 | -73.541 | 0.001 | 0.830 | -19.323 | 0.087 | 0.208 | -50.055 |
| **PO₄³⁻** | 0.003 | 0.484 | -9.238 | 0.008 | 0.346 | 28.209 | 1 | 0 | 0 | 1 | 0 | 0 |
| **SO₄²^-^** | 0.449 | 0.063 | -28.341 | 0.017 | 0.118 | -104.738 | 0.001 | 0.900 | -102.787 | 0.038 | 0.349 | 107.028 |
| **O_2_** | 0.001 | 0.561 | 44.879 | 0.549 | 0.163 | 97.969 | 0.015 | 0.382 | -57.447 | 0.002 | 0.583 | 73.316 |
| **T** | 0.001 | 0.872 | -131.472 | 0.106 | 0.143 | -154.699 | 0.001 | 0.765 | 107.038 | 0.031 | 0.358 | -97.152 |
| **Turb** | 0.053 | 0.197 | 69.406 | 0.051 | 0.196 | -167.028 | 0.003 | 0.554 | -57.239 | 0.05 | 0.290 | 68.500 |
| **pH** | 0.01 | 0.377 | -89.647 | 0.024 | 0.256 | 148.657 | 0.052 | 0.281 | -81.150 | 0.993 | 0.012 | 81.729 |
| **EC** | 0.001 | 0.478 | -143.435 | 0.702 | 0.108 | -88.756 | 0.005 | 0.544 | -123.784 | 0.001 | 0.593 | -143.680 |

**Table S5** ASVs that resulted enriched in the differential abundances analysis performed with ANCOM-BC2 when comparing the samples of the extensive site of the Perlenbach against the one from the forest at the two sampling times.

| **ASV** | **Biofilms** | **Sampling time** | **Enriched in** | **Log fold change** | ***P* adjusted value** | **Lowest taxon** |
| --- | --- | --- | --- | --- | --- | --- |
| ASV7 | DB | T1 | FP | 3.150 | 2.200E-05 | *Alteraurantiacibacter* |
| ASV138 | MB | T2 | FP | 1.636 | 4.060E-02 | *Polymorphobacter* |
| ASV391 | MB | T2 | EP | -2.694 | 3.698E-02 | Hyphomicrobiales |
| ASV459 | MB | T2 | EP | -2.420 | 1.313E-02 | Actinobacteria |

**Table S6** Bins selected in the iCAMP analysis with their top taxon and their contribution to the relative importance (%) of the bacterial community assembly processes. Highlighted in bold the taxa in the bins driving deterministic processes, which were also detected as enriched by agricultural land use in the differential abundance analyses.

| **Sample** | **ST** | **Site** | **Process** | **Bin** | **Taxa** | **Relative importance (%)** |
| --- | --- | --- | --- | --- | --- | --- |
| DB | T1 | FO | DL | bin55 | *Actinoplanes* | 3.75 |
| DB | T1 | FO | DL | bin56 | *Pseudonocardia* | 3.22 |
| DB | T1 | IO | DL | bin83 | *Sphaerotilus* | 3.17 |
| DB | T1 | FO | DL | bin28 | *Blastocatella* | 2.51 |
| DB | T1 | FO | DL | bin52 | *Actinobacteria* | 1.98 |
| DB | T1 | IO | DL | bin70 | *Crenothrix* | 1.73 |
| DB | T1 | FO | DL | bin74 | *Pseudomonas* | 1.72 |
| DB | T1 | FO | DL | bin51 | *Rhodoluna* | 1.32 |
| DB | T1 | FO | DL | bin49 | *Candidatus Planktophila* | 1.17 |
| DB | T1 | FO | DL | bin48 | *Microtrichaceae* | 1.11 |
| DB | T1 | EO | DR | bin80 | *Leptothrix* | 6.83 |
| DB | T1 | EO | DR | bin12 | *Ferruginibacter* | 4.98 |
| DB | T1 | IO | DR | bin12 | *Ferruginibacter* | 4.05 |
| DB | T1 | IO | DR | bin72 | *Steroidobacteraceae* | 3.56 |
| DB | T1 | FO | DR | bin77 | *Parvibium* | 3.08 |
| DB | T1 | FO | DR | bin72 | *Steroidobacteraceae* | 3.04 |
| DB | T1 | EO | DR | bin5 | *Candidatus Amoebophilus* | 2.80 |
| DB | T1 | IO | DR | bin80 | *Leptothrix* | 2.64 |
| DB | T1 | FO | DR | bin15 | *Flavihumibacter* | 2.50 |
| DB | T1 | FO | DR | bin43 | *Ilumatobacteraceae* | 2.49 |
| DB | T1 | IO | DR | bin83 | *Sphaerotilus* | 2.42 |
| DB | T1 | FO | DR | bin80 | *Leptothrix* | 2.35 |
| DB | T1 | IO | DR | bin75 | *Burkholderiales;SC-I-84* | 2.28 |
| DB | T1 | IO | DR | bin43 | *Ilumatobacteraceae* | 1.85 |
| DB | T1 | IO | DR | bin79 | *Sutterellaceae;AAP99* | 1.84 |
| DB | T1 | EO | DR | bin78 | *Methylotenera* | 1.64 |
| DB | T1 | FO | DR | bin78 | *Methylotenera* | 1.62 |
| DB | T1 | FO | DR | bin82 | *Rhodoferax* | 1.58 |
| DB | T1 | FO | DR | bin44 | *Ilumatobacter* | 1.50 |
| DB | T1 | FO | DR | bin46 | *Ilumatobacteraceae* | 1.46 |
| DB | T1 | IO | DR | bin44 | *Ilumatobacter* | 1.40 |
| DB | T1 | FO | DR | bin12 | *Ferruginibacter* | 1.36 |
| DB | T1 | IO | DR | bin84 | *Hydrogenophaga* | 1.35 |
| DB | T1 | FO | DR | bin3 | *Saprospiraceae* | 1.30 |
| DB | T1 | FO | DR | bin47 | *Acidimicrobiia* | 1.29 |
| DB | T1 | IO | DR | bin49 | *Candidatus Planktophila* | 1.29 |
| DB | T1 | EO | DR | bin79 | *Sutterellaceae;AAP99* | 1.26 |
| DB | T1 | IO | DR | bin3 | *Saprospiraceae* | 1.21 |
| DB | T1 | FO | DR | bin36 | *Gemmatimonas* | 1.15 |
| DB | T1 | FO | DR | bin5 | *Candidatus Amoebophilus* | 1.13 |
| DB | T1 | FO | DR | bin83 | *Sphaerotilus* | 1.11 |
| DB | T1 | IO | DR | bin5 | *Candidatus Amoebophilus* | 1.03 |
| DB | T1 | FO | DR | bin30 | *Deinococcaceae* | 1.02 |
| DB | T1 | IO | DR | bin78 | *Methylotenera* | 1.00 |
| DB | T1 | EO | HD | bin80 | *Leptothrix* | 4.84 |
| DB | T1 | EO | HD | bin15 | *Flavihumibacter* | 2.19 |
| DB | T1 | FO | HD | bin80 | *Leptothrix* | 1.51 |
| DB | T1 | FO | HD | bin30 | *Deinococcaceae* | 1.29 |
| DB | T1 | EO | HD | bin72 | *Steroidobacteraceae* | 1.17 |
| DB | T1 | FO | HD | bin36 | *Gemmatimonas* | 1.17 |
| DB | T1 | FO | HD | bin47 | *Acidimicrobiia* | 1.09 |
| DB | T1 | EO | HoS | bin36 | ***Gemmatimonas*** | 11.68 |
| DB | T1 | EO | HoS | bin30 | ***Deinococcaceae*** | 11.56 |
| DB | T1 | IO | HoS | bin80 | ***Leptothrix*** | 10.25 |
| DB | T1 | EO | HoS | bin47 | ***Acidimicrobiia*** | 9.66 |
| DB | T1 | EO | HoS | bin77 | ***Parvibium*** | 8.72 |
| DB | T1 | IO | HoS | bin82 | ***Rhodoferax*** | 7.49 |
| DB | T1 | IO | HoS | bin36 | ***Gemmatimonas*** | 5.53 |
| DB | T1 | EO | HoS | bin44 | ***Ilumatobacter*** | 3.96 |
| DB | T1 | IO | HoS | bin47 | ***Acidimicrobiia*** | 3.89 |
| DB | T1 | IO | HoS | bin15 | *Flavihumibacter* | 3.84 |
| DB | T1 | EO | HoS | bin3 | *Saprospiraceae* | 3.24 |
| DB | T1 | IO | HoS | bin77 | *Parvibium* | 3.20 |
| DB | T1 | EO | HoS | bin82 | ***Rhodoferax*** | 2.81 |
| DB | T1 | EO | HoS | bin28 | *Blastocatella* | 2.69 |
| DB | T1 | FO | HoS | bin75 | *Burkholderiales* | 2.16 |
| DB | T1 | FO | HoS | bin47 | *Acidimicrobiia* | 1.63 |
| DB | T1 | FO | HoS | bin80 | *Leptothrix* | 1.48 |
| DB | T1 | EO | HoS | bin13 | *Ferruginibacter* | 1.09 |
| DB | T2 | EO | DL | bin119 | *Oxalobacteraceae* | 2.70 |
| DB | T2 | FO | DL | bin63 | *Ilumatobacteraceae* | 1.52 |
| DB | T2 | IO | DL | bin120 | *Dechloromonas* | 1.23 |
| DB | T2 | FO | DR | bin102 | *Spongiibacteraceae* | 9.69 |
| DB | T2 | EO | DR | bin111 | *Rhizobacter* | 8.68 |
| DB | T2 | EO | DR | bin15 | *Flavobacterium* | 5.83 |
| DB | T2 | FO | DR | bin104 | *Steroidobacteraceae* | 5.42 |
| DB | T2 | EO | DR | bin9 | *Candidatus Amoebophilus* | 4.54 |
| DB | T2 | IO | DR | bin113 | *Leptothrix* | 3.59 |
| DB | T2 | EO | DR | bin112 | *Leptothrix* | 3.27 |
| DB | T2 | FO | DR | bin108 | *Methylophilaceae* | 3.11 |
| DB | T2 | EO | DR | bin33 | *Arcicella* | 2.93 |
| DB | T2 | EO | DR | bin19 | *Ferruginibacter* | 2.68 |
| DB | T2 | EO | DR | bin103 | *Agitococcus* | 2.52 |
| DB | T2 | FO | DR | bin100 | *Gammaproteobacteria;R7C24* | 2.29 |
| DB | T2 | IO | DR | bin69 | *Intrasporangiaceae* | 1.74 |
| DB | T2 | IO | DR | bin15 | *Flavobacterium* | 1.49 |
| DB | T2 | IO | DR | bin108 | *Methylophilaceae* | 1.49 |
| DB | T2 | FO | DR | bin103 | *Agitococcus* | 1.44 |
| DB | T2 | FO | DR | bin79 | *Vicinamibacteria* | 1.37 |
| DB | T2 | FO | DR | bin69 | *Intrasporangiaceae* | 1.34 |
| DB | T2 | EO | DR | bin4 | *Haliscomenobacter* | 1.33 |
| DB | T2 | EO | DR | bin119 | *Oxalobacteraceae* | 1.29 |
| DB | T2 | IO | DR | bin120 | *Dechloromonas* | 1.22 |
| DB | T2 | FO | DR | bin8 | *Lacihabitans* | 1.21 |
| DB | T2 | IO | DR | bin79 | *Vicinamibacteria* | 1.19 |
| DB | T2 | IO | DR | bin119 | *Oxalobacteraceae* | 1.18 |
| DB | T2 | IO | DR | bin112 | *Leptothrix* | 1.18 |
| DB | T2 | IO | DR | bin102 | *Spongiibacteraceae* | 1.15 |
| DB | T2 | EO | DR | bin22 | *Flavihumibacter* | 1.11 |
| DB | T2 | IO | DR | bin111 | *Rhizobacter* | 1.07 |
| DB | T2 | EO | HD | bin117 | *Arenimonas* | 3.97 |
| DB | T2 | IO | HD | bin102 | *Spongiibacteraceae* | 3.57 |
| DB | T2 | FO | HD | bin108 | *Methylophilaceae* | 1.99 |
| DB | T2 | FO | HD | bin111 | *Rhizobacter* | 1.96 |
| DB | T2 | FO | HD | bin112 | *Leptothrix* | 1.69 |
| DB | T2 | EO | HD | bin108 | *Methylophilaceae* | 1.57 |
| DB | T2 | IO | HD | bin108 | *Methylophilaceae* | 1.55 |
| DB | T2 | IO | HD | bin111 | *Rhizobacter* | 1.46 |
| DB | T2 | EO | HD | bin102 | *Spongiibacteraceae* | 1.34 |
| DB | T2 | FO | HD | bin102 | *Spongiibacteraceae* | 1.07 |
| DB | T2 | EO | HeS | bin18 | *Sphingobacteriales* | 1.05 |
| DB | T2 | IO | HoS | bin115 | *Rhodoferax* | 13.29 |
| DB | T2 | EO | HoS | bin115 | ***Rhodoferax*** | 10.38 |
| DB | T2 | FO | HoS | bin115 | *Rhodoferax* | 5.70 |
| DB | T2 | FO | HoS | bin105 | *Burkholderiales;SC-I-85* | 3.99 |
| DB | T2 | EO | HoS | bin52 | ***Gemmatimonas*** | 3.83 |
| DB | T2 | IO | HoS | bin33 | ***Arcicella*** | 3.73 |
| DB | T2 | EO | HoS | bin16 | ***Flavobacterium*** | 3.63 |
| DB | T2 | IO | HoS | bin105 | *Burkholderiales;SC-I-84* | 3.18 |
| DB | T2 | EO | HoS | bin8 | *Lacihabitans* | 2.87 |
| DB | T2 | FO | HoS | bin117 | *Arenimonas* | 2.86 |
| DB | T2 | EO | HoS | bin13 | *Fluviicola* | 2.85 |
| DB | T2 | IO | HoS | bin104 | ***Steroidobacteraceae*** | 2.68 |
| DB | T2 | IO | HoS | bin117 | *Arenimonas* | 2.51 |
| DB | T2 | IO | HoS | bin8 | ***Lacihabitans*** | 2.35 |
| DB | T2 | IO | HoS | bin16 | *Flavobacterium* | 2.15 |
| DB | T2 | FO | HoS | bin16 | *Flavobacterium* | 2.13 |
| DB | T2 | FO | HoS | bin113 | *Leptothrix* | 2.06 |
| DB | T2 | FO | HoS | bin11 | *Spirosomaceae* | 2.05 |
| DB | T2 | FO | HoS | bin4 | *Haliscomenobacter* | 2.00 |
| DB | T2 | IO | HoS | bin11 | *Spirosomaceae* | 1.71 |
| DB | T2 | IO | HoS | bin70 | *Longivirga* | 1.68 |
| DB | T2 | IO | HoS | bin99 | *Methylobacter* | 1.68 |
| DB | T2 | FO | HoS | bin22 | *Flavihumibacter* | 1.64 |
| DB | T2 | FO | HoS | bin19 | *Ferruginibacter* | 1.63 |
| DB | T2 | IO | HoS | bin19 | *Ferruginibacter* | 1.52 |
| DB | T2 | IO | HoS | bin103 | *Agitococcus* | 1.50 |
| DB | T2 | EO | HoS | bin65 | *Acidimicrobiia* | 1.32 |
| DB | T2 | EO | HoS | bin19 | *Ferruginibacter* | 1.27 |
| DB | T2 | IO | HoS | bin102 | *Spongiibacteraceae* | 1.19 |
| DB | T2 | FO | HoS | bin78 | *Nannocystis* | 1.16 |
| DB | T2 | FO | HoS | bin107 | *Parvibium* | 1.03 |
| DB | T2 | EO | HoS | bin107 | *Parvibium* | 1.03 |
| MB | T1 | IO | DL | bin104 | *Nitrospira* | 5.30 |
| MB | T1 | IO | DL | bin97 | *Methyloglobulus* | 2.38 |
| MB | T1 | IO | DL | bin78 | *Vicinamibacteria; Subgroup17* | 1.46 |
| MB | T1 | IO | DL | bin60 | *Clostridium* | 1.17 |
| MB | T1 | IO | DL | bin90 | *Anaeromyxobacter* | 1.15 |
| MB | T1 | IO | DL | bin54 | *Haliangium* | 1.11 |
| MB | T1 | IO | DL | bin36 | *Blastocatellia* | 1.04 |
| MB | T1 | EO | DR | bin12 | *Ferruginibacter* | 4.11 |
| MB | T1 | EO | DR | bin116 | *Ellin6067* | 3.37 |
| MB | T1 | EO | DR | bin114 | *Schlegelella* | 2.83 |
| MB | T1 | EO | DR | bin23 | *Arcicella* | 2.76 |
| MB | T1 | EO | DR | bin35 | *Blastocatella* | 2.04 |
| MB | T1 | EO | DR | bin13 | *Ferruginibacter* | 2.00 |
| MB | T1 | EO | DR | bin72 | *Nocardioides* | 1.91 |
| MB | T1 | EO | DR | bin9 | *Flavobacterium* | 1.69 |
| MB | T1 | EO | DR | bin15 | *Chitinophagaceae* | 1.57 |
| MB | T1 | EO | DR | bin107 | *Parvibium* | 1.17 |
| MB | T1 | EO | DR | bin68 | *Microbacteriaceae* | 1.05 |
| MB | T1 | EO | DR | bin5 | *Lacihabitans* | 1.00 |
| MB | T1 | FO | DR | bin40 | *Deinococcaceae* | 12.11 |
| MB | T1 | FO | DR | bin40 | *Deinococcaceae* | 7.80 |
| MB | T1 | FO | DR | bin113 | *Leptothrix* | 7.71 |
| MB | T1 | FO | DR | bin13 | *Ferruginibacter* | 6.84 |
| MB | T1 | FO | DR | bin107 | *Parvibium* | 5.63 |
| MB | T1 | FO | DR | bin65 | *Actinobacteriota* | 4.69 |
| MB | T1 | FO | DR | bin68 | *Microbacteriaceae* | 3.25 |
| MB | T1 | FO | DR | bin118 | *Lysobacter* | 2.99 |
| MB | T1 | FO | DR | bin5 | *Lacihabitans* | 2.85 |
| MB | T1 | FO | DR | bin100 | *Aeromonas* | 2.48 |
| MB | T1 | FO | DR | bin61 | *Ilumatobacteraceae* | 2.21 |
| MB | T1 | FO | DR | bin115 | *Rhodoferax* | 1.98 |
| MB | T1 | FO | DR | bin67 | *Candidatus Planktophila* | 1.87 |
| MB | T1 | FO | DR | bin109 | *Polynucleobacter* | 1.71 |
| MB | T1 | FO | DR | bin15 | *Chitinophagaceae* | 1.59 |
| MB | T1 | FO | DR | bin12 | *Ferruginibacter* | 1.29 |
| MB | T1 | FO | DR | bin65 | *Actinobacteriota* | 1.21 |
| MB | T1 | FO | DR | bin23 | *Arcicella* | 1.07 |
| MB | T1 | FO | DR | bin103 | *Pseudomonas* | 1.01 |
| MB | T1 | IO | DR | bin67 | *Candidatus Planktophila* | 2.91 |
| MB | T1 | IO | DR | bin104 | *Nitrospira* | 2.12 |
| MB | T1 | IO | DR | bin48 | *Gemmatimonas* | 1.82 |
| MB | T1 | IO | DR | bin96 | *Methyloglobulus* | 1.69 |
| MB | T1 | IO | DR | bin72 | *Nocardioides* | 1.49 |
| MB | T1 | IO | DR | bin113 | *Leptothrix* | 1.14 |
| MB | T1 | EO | HD | bin113 | *Leptothrix* | 6.85 |
| MB | T1 | EO | HD | bin40 | *Deinococcaceae* | 1.77 |
| MB | T1 | EO | HD | bin116 | *Ellin6067* | 1.60 |
| MB | T1 | EO | HD | bin107 | *Parvibium* | 1.27 |
| MB | T1 | IO | HD | bin113 | *Leptothrix* | 2.93 |
| MB | T1 | IO | HD | bin115 | *Rhodoferax* | 1.80 |
| MB | T1 | FO | HeS | bin116 | *Ellin6067* | 1.15 |
| MB | T1 | IO | HeS | bin96 | *Methyloglobulus* | 4.39 |
| MB | T1 | EO | HoS | bin115 | *Rhodoferax* | 7.26 |
| MB | T1 | EO | HoS | bin40 | *Deinococcaceae* | 7.10 |
| MB | T1 | EO | HoS | bin65 | *Actinobacteriota* | 5.02 |
| MB | T1 | EO | HoS | bin111 | *Sutterellaceae;AAP99* | 4.45 |
| MB | T1 | EO | HoS | bin41 | *Deinococcaceae* | 4.07 |
| MB | T1 | EO | HoS | bin118 | ***Lysobacter*** | 3.74 |
| MB | T1 | EO | HoS | bin48 | *Gemmatimonas* | 3.01 |
| MB | T1 | EO | HoS | bin119 | *Dechloromonas* | 1.56 |
| MB | T1 | EO | HoS | bin72 | *Nocardioides* | 1.07 |
| MB | T1 | IO | HoS | bin113 | *Leptothrix* | 5.22 |
| MB | T1 | IO | HoS | bin41 | *Deinococcaceae* | 1.88 |
| MB | T1 | IO | HoS | bin105 | *Burkholderiales;SC-I-84* | 1.28 |
| MB | T1 | IO | HoS | bin80 | *Geothrix* | 1.08 |
| MB | T1 | IO | HoS | bin119 | *Dechloromonas* | 1.05 |
| MB | T2 | FO | DL | bin182 | *Gammaproteobacteria;R7C24* | 2.03 |
| MB | T2 | FO | DL | bin79 | *Deinococcaceae* | 2.00 |
| MB | T2 | IO | DL | bin206 | *Leptothrix* | 1.77 |
| MB | T2 | IO | DL | bin190 | *Nitrospira* | 1.46 |
| MB | T2 | IO | DL | bin176 | *Methyloglobulus* | 1.20 |
| MB | T2 | EO | DL | bin108 | *Gaiella* | 1.01 |
| MB | T2 | IO | DR | bin190 | *Nitrospira* | 4.45 |
| MB | T2 | FO | DR | bin205 | *Leptothrix* | 3.60 |
| MB | T2 | IO | DR | bin176 | *Methyloglobulus* | 3.54 |
| MB | T2 | FO | DR | bin189 | *Steroidobacteraceae* | 2.45 |
| MB | T2 | IO | DR | bin205 | *Leptothrix* | 2.22 |
| MB | T2 | FO | DR | bin72 | *Blastocatellaceae;JGI 0001001-H03* | 1.95 |
| MB | T2 | FO | DR | bin31 | *Ferruginibacter* | 1.86 |
| MB | T2 | FO | DR | bin36 | *Flavihumibacter* | 1.86 |
| MB | T2 | IO | DR | bin206 | *Leptothrix* | 1.60 |
| MB | T2 | FO | DR | bin212 | *Sphaerotilus* | 1.54 |
| MB | T2 | EO | DR | bin200 | *Sulfuritalea* | 1.48 |
| MB | T2 | FO | DR | bin79 | *Deinococcaceae* | 1.41 |
| MB | T2 | FO | DR | bin186 | *Halieaceae* | 1.38 |
| MB | T2 | FO | DR | bin216 | *Arenimonas* | 1.36 |
| MB | T2 | FO | DR | bin211 | *Limnohabitans* | 1.34 |
| MB | T2 | EO | DR | bin112 | *Ilumatobacteraceae;CL500-29 marine group* | 1.32 |
| MB | T2 | IO | DR | bin188 | *Pseudomonas* | 1.29 |
| MB | T2 | FO | DR | bin198 | *Methylotenera* | 1.22 |
| MB | T2 | IO | DR | bin186 | *Halieaceae* | 1.21 |
| MB | T2 | EO | DR | bin124 | *Longivirga* | 1.18 |
| MB | T2 | FO | DR | bin28 | *Flavobacterium* | 1.17 |
| MB | T2 | IO | DR | bin72 | *Blastocatellaceae;JGI 0001001-H03* | 1.17 |
| MB | T2 | EO | DR | bin123 | *Actinobacteria;PeM15* | 1.13 |
| MB | T2 | FO | DR | bin197 | *Sutterellaceae* | 1.10 |
| MB | T2 | FO | DR | bin214 | *Limnohabitans* | 1.09 |
| MB | T2 | FO | DR | bin120 | *Microbacteriaceae* | 1.08 |
| MB | T2 | FO | DR | bin54 | *Arcicella* | 1.03 |
| MB | T2 | IO | HD | bin205 | *Leptothrix* | 1.44 |
| MB | T2 | EO | HD | bin216 | *Arenimonas* | 1.04 |
| MB | T2 | IO | HeS | bin177 | *Methyloglobulus* | 1.79 |
| MB | T2 | FO | HoS | bin210 | *Rhodoferax* | 7.75 |
| MB | T2 | EO | HoS | bin210 | *Rhodoferax* | 5.18 |
| MB | T2 | IO | HoS | bin210 | *Rhodoferax* | 4.01 |
| MB | T2 | EO | HoS | bin31 | *Ferruginibacter* | 3.58 |
| MB | T2 | EO | HoS | bin189 | *Steroidobacteraceae* | 3.53 |
| MB | T2 | IO | HoS | bin212 | *Sphaerotilus* | 2.89 |
| MB | T2 | EO | HoS | bin186 | *Halieaceae* | 2.69 |
| MB | T2 | EO | HoS | bin191 | *Burkholderiales;SC-I-84* | 2.60 |
| MB | T2 | EO | HoS | bin192 | *Burkholderiales;SC-I-86* | 2.47 |
| MB | T2 | EO | HoS | bin205 | *Leptothrix* | 1.94 |
| MB | T2 | IO | HoS | bin11 | *Lacihabitans* | 1.70 |
| MB | T2 | FO | HoS | bin14 | *Spirosomaceae* | 1.69 |
| MB | T2 | EO | HoS | bin36 | *Flavihumibacter* | 1.63 |
| MB | T2 | FO | HoS | bin11 | *Lacihabitans* | 1.58 |
| MB | T2 | EO | HoS | bin28 | *Flavobacterium* | 1.48 |
| MB | T2 | IO | HoS | bin28 | *Flavobacterium* | 1.35 |
| MB | T2 | IO | HoS | bin36 | *Flavihumibacter* | 1.31 |
| MB | T2 | FO | HoS | bin191 | *Burkholderiales;SC-I-84* | 1.30 |
| MB | T2 | EO | HoS | bin190 | *Nitrospira* | 1.28 |
| MB | T2 | EO | HoS | bin14 | *Spirosomaceae* | 1.26 |
| MB | T2 | IO | HoS | bin197 | *Sutterellaceae* | 1.23 |
| MB | T2 | FO | HoS | bin192 | *Burkholderiales;SC-I-87* | 1.23 |
| MB | T2 | EO | HoS | bin39 | *Dinghuibacter* | 1.23 |
| MB | T2 | FO | HoS | bin185 | *Moraxellaceae* | 1.11 |
| MB | T2 | EO | HoS | bin11 | *Lacihabitans* | 1.11 |
| MB | T2 | IO | HoS | bin192 | *Burkholderiales;SC-I-85* | 1.09 |

**Table S7** Relative importance of processes in the bacterial community assembly of developing (DB) and mature biofilms (MB) across the sites of the Otterbach (EO, IO and FO) and the Perlenbach stream (EP and FP) at the summer (T1) and autumn (T2) sampling time.

| **CAA (%)** | | **T1** | | | | | **T2** | | | | |
| --- | --- | --- | --- | --- | --- | --- | --- | --- | --- | --- | --- |
|  |  | **HeS** | **HoS** | **DL** | **HD** | **DR** | **HeS** | **HoS** | **DL** | **HD** | **DR** |
| DB | FO | 1.3 | 14 | 38.1 | 5.8 | 40.8 | 0.3 | 31.8 | 12.1 | 9.9 | 45.9 |
|  | EO | 0 | 59.6 | 1.4 | 8.8 | 30.2 | 1.2 | 31.3 | 3.5 | 10.9 | 53.1 |
|  | IO | 0.1 | 40.5 | 12.8 | 3.4 | 43.2 | 0.3 | 42.3 | 8.5 | 10.8 | 38.2 |
| MB | FO | 2 | 23.4 | 3.33 | 2.7 | 68.6 | 1.5 | 22.2 | 13.4 | 1.9 | 61 |
|  | EO | 0.2 | 42.4 | 3.7 | 13.7 | 40 | 0.5 | 39 | 12 | 7.1 | 41.4 |
|  | IO | 5.9 | 23.1 | 33 | 9.8 | 28.2 | 1.9 | 28.1 | 14.6 | 6.5 | 48.9 |
| DB | FP | 0.8 | 39.6 | 5.6 | 17.9 | 36.1 | 1 | 39.4 | 2.7 | 6.2 | 50.7 |
|  | EP | 0.1 | 24.2 | 19.7 | 13.7 | 42.3 | 0.5 | 36.6 | 8.4 | 11.8 | 42.6 |
| MB | FP | 4.4 | 19.7 | 42.4 | 1.8 | 31.7 | 1.3 | 27 | 13 | 6.7 | 52.1 |
|  | EP | 0 | 35.2 | 0.9 | 13.7 | 50.2 | 1.2 | 26.8 | 31.1 | 4 | 36.8 |

**Table S8** Properties of bacterial co-occurrence networks from the developing biofilms (DB) and mature biofilms (MB) samples from the agricultural sites of the Otterbach and Perlenbach.

| **Co-occurrence network properties** | **Otterbach** | | | | | |
| --- | --- | --- | --- | --- | --- | --- |
|  | **DB** | | | **MB** | | |
|  | **IO** | **EO** | **FO** | **IO** | **EO** | **FO** |
| Number of components | 1 | 13 | 2 | 1 | 1 | 7 |
| Clustering coefficient | 0.104 | 0.140 | 0.120 | 0.122 | 0.083 | 0.097 |
| Modularity | 0.586 | 0.774 | 0.674 | 0.561 | 0.613 | 0.795 |
| Positive edge percentage | 58.719 | 68.224 | 63.750 | 62.521 | 63.576 | 70.289 |
| Edge density | 0.023 | 0.020 | 0.020 | 0.020 | 0.022 | 0.017 |
| Natural connectivity | 0.008 | 0.011 | 0.008 | 0.005 | 0.007 | 0.009 |
| Number of nodes | 157 | 97 | 155 | 246 | 168 | 127 |
| **Co-occurrence network properties** | **Perlenbach** | | | |  |  |
|  | **DB** | | **MB** | |  |  |
|  | **EP** | **FP** | **EP** | **FP** |  |  |
| Number of components | 16 | 1 | 1 | 3 |  |  |
| Clustering coefficient | 0.061 | 0.120 | 0.0866 | 0.127 |  |  |
| Modularity | 0.793 | 0.621 | 0.65 | 0.586 |  |  |
| Positive edge percentage | 73.248 | 69.140 | 67.419 | 64.966 |  |  |
| Edge density | 0.014 | 0.023 | 0.0199 | 0.022 |  |  |
| Natural connectivity | 0.007 | 0.008 | 0.007 | 0.007 |  |  |
| Number of nodes | 148 | 151 | 177 | 165 |  |  |

**Table S9** Main module hubs taxa of the co-occurrence networks from the Otterbach sites under agricultural land use, intensive (IO) and extensive (EO), in developing biofilm (DB) and mature biofilm (MB) samples.

| **Sample type** | **Site** | **Main module hub taxa** |
| --- | --- | --- |
| DB | IO | *Blastocatella*, *Candidatus Amoebophilu*s, *Dinghuibacter*, *Georgfuchsia, Nitrospira*, *Sulfuritalea*, *Syntrophorhabdus*, *Truepera* |
|  | EO | *Candidatus Planktophila* *Cloacibacterium*, *Gemmatimonas*, *Pantoea*, *Parvibium*, *Serratia* |
|  | FO | *Agitococcus, Arcicella, Aurantisolimonas, Galbitaliea, MM1, Nannocystis, Sulfuritalea, Thiobacillus* |
| MB | IO | *Arthrobacter*, *Buchnera*, CL500-29 marine group, *Desulfobulbus*, *Haliangium*, IMCC26207, *Legionella*, *Leptothrix*, *Massilia*, *Microlunatus, Phaeodactylibacter*, *Solitalea*, *Sorangium* |
|  | EO | *Aurantimicrobium*, *Dinghuibacter*, *Gaiella* OLB12, *Oligoflexus*, OM60(NOR5) clade, *Pseudomonas*, *Tahibacter,* *Truepera* |
|  | FO | *Aurantisolimonas*, *Flavitalea*, *MND1*, *Nannocystis*, *Ohtaekwangia*, *Sphaerotilus*, *Thiobacillus* |
| **DB** | EP | *Aureispira, BD1-7 clade, BIyi10, Chitinibacter, hgcI clade, Methyloglobulus, Polynucleobacter, Rhodoluna* |
|  | FP | *Aridibacter, Bryobacter, Edaphobaculum, Ferruginibacter, Piscinibacter, Rhodoferax, Segetibacter, Terrimonas* |
| **MB** | EP | *Actinoplanes, Aeromicrobium, Lysinimonas, Marmoricola, Massilia, Mucilaginibacter, Quatrionicoccus, Spirochaeta, Tolumonas* |
|  | FP | *Dechloromonas, Gemmatimonas, Malikia, Massilia, Methyloglobulus, Nitrospira, Rhodoferax, Rurumicrobium, Sulfuritalea* |

**Supplementary figures**


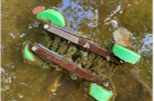


**Fig. S1.** One of the installed coupon sampling devices with the microscope glass slides.


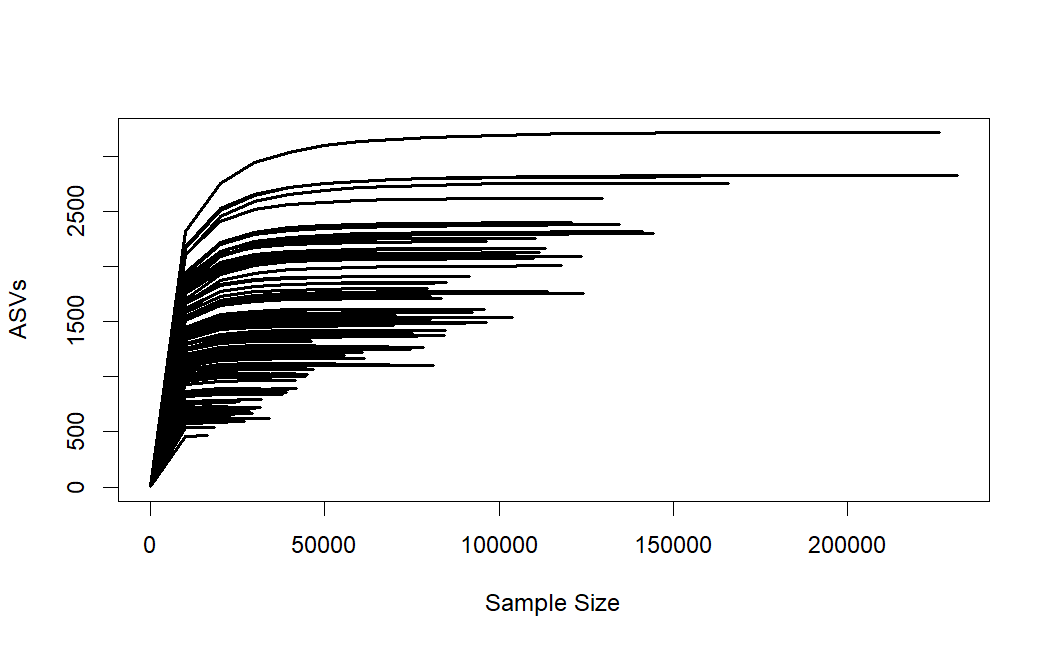


**Fig. S2.** Rarefaction curves displaying the number of ASVs detected per sample as a function of sequencing depth.

**Fig. S3.** Beta-dispersion of bacterial communities from developing (DB) and mature (MB) biofilms across sites of the Otterbach (A and B) and Perlenbach (C and D) at the summer (T1) and autumn (T2) sampling times. Boxplots represent distances to group centroids based on Bray–Curtis dissimilarities. Differences among sites were assessed using the Kruskal–Wallis test followed by pairwise Wilcoxon rank-sum tests. P values were corrected using the false discovery rate (fdr) method.


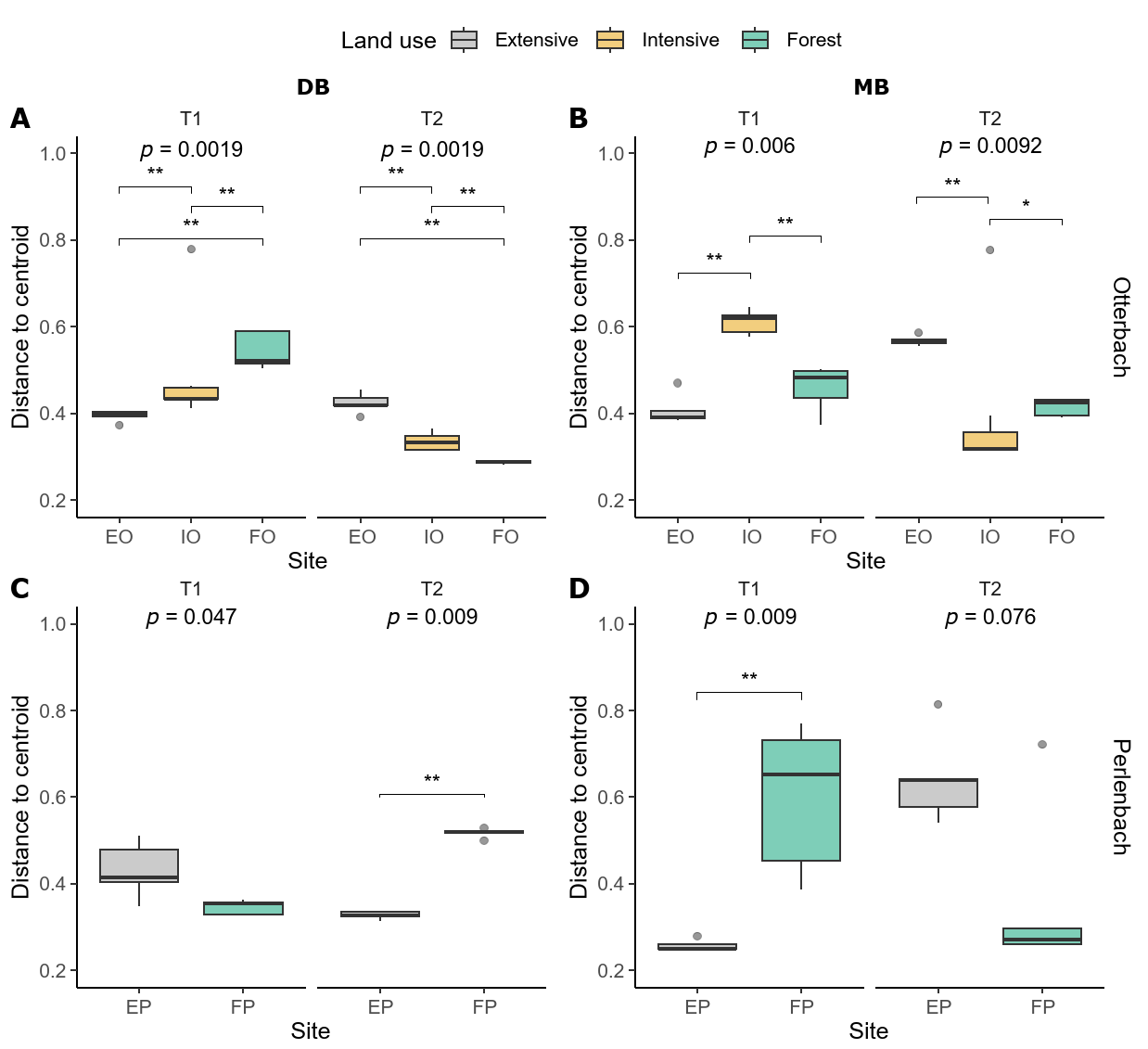

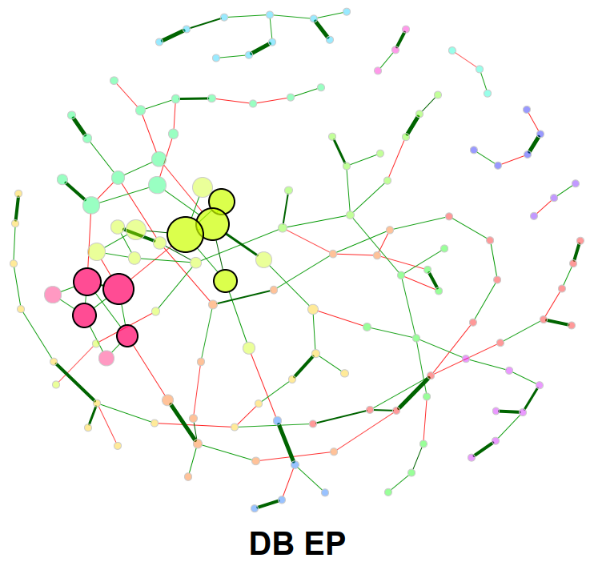

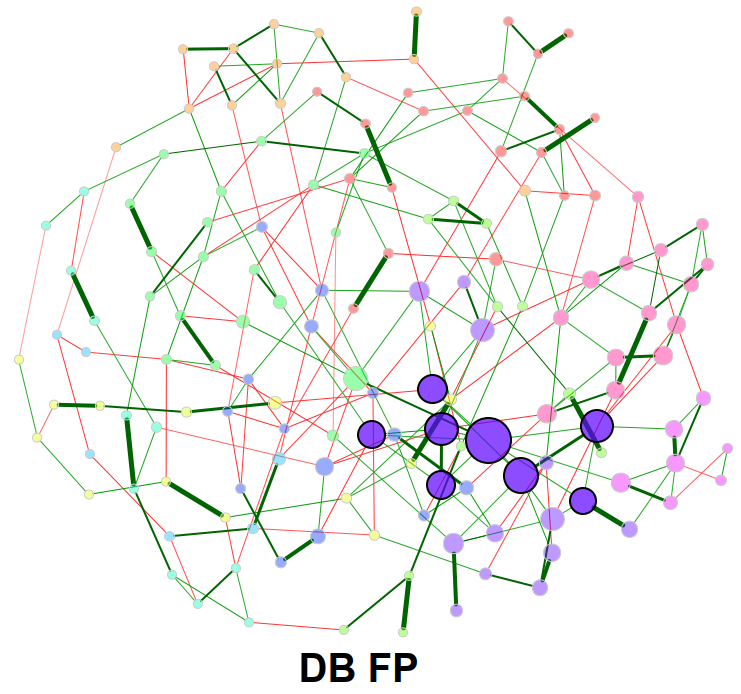

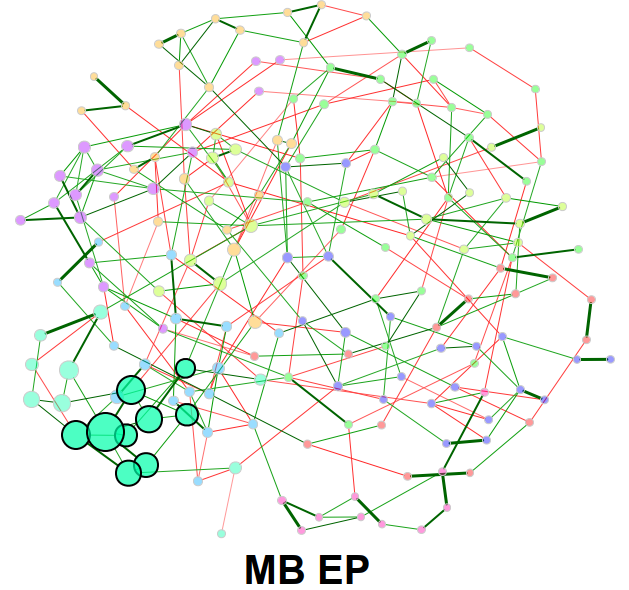

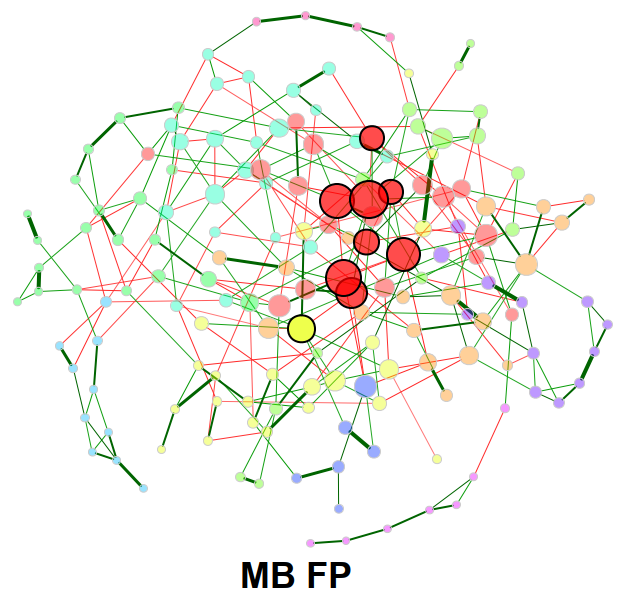


**Fig. S4.** Co-occurrence networks of bacterial communities from developing (DB) and mature biofilm (MB) samples from the Perlenbach sites: extensive grassland (EP) and forest (FP). Despite significant beta diversity differences between sampling times, the networks were constructed combining samples from the two sampling times (n=10). Nodes are coloured according to cluster.

**Fig. S6.** Monthly accumulated rainfall (mm) in 2023, displayed as barplots, with a line indicating average daily rainfall (mm). The red arrows indicate when the sampling events took place.


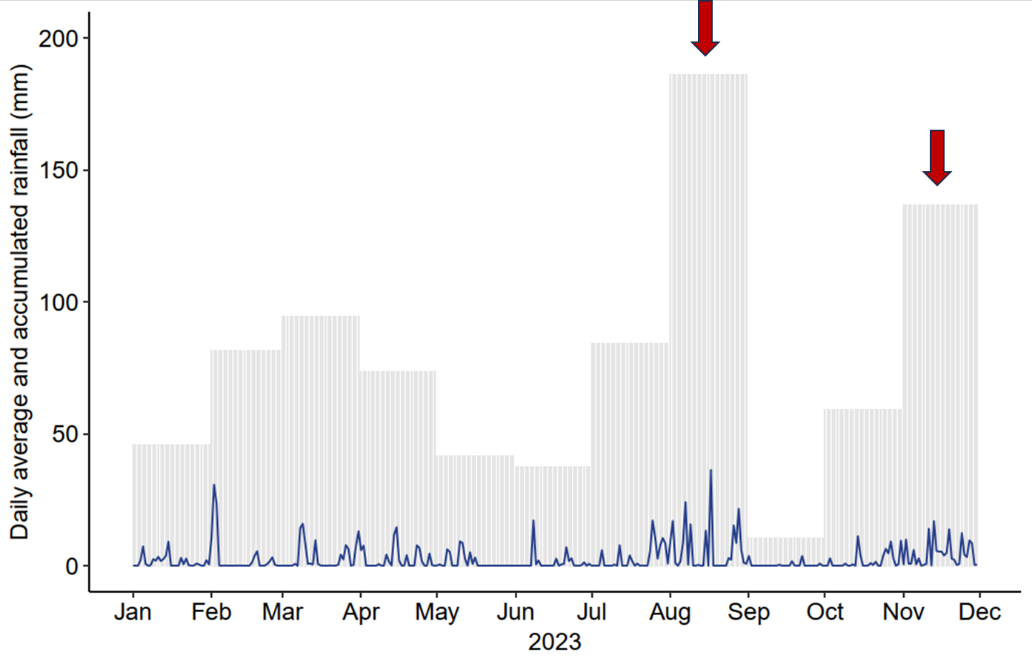


**Fig. S5.** Daily average concentration of dissolved organic carbon (DOC) in mg/L measured from August to the end of November at the intensive farming (IO) and the forest (FO) sites of the Otterbach.


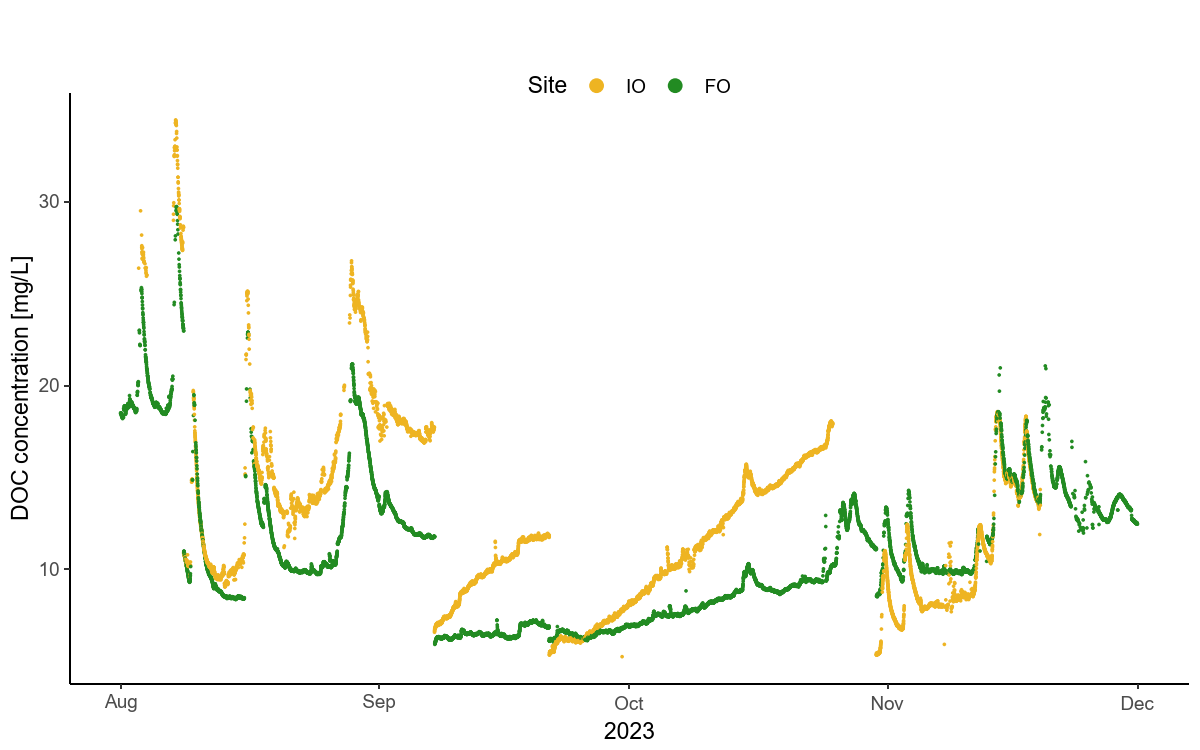

Supplement: Supplementary file 1 — Supplementary Material 1 [file 40793_2025_837_MOESM1_ESM.docx]
